# Evaluation of the sugar-sweetened beverage tax in Oakland, United States, 2015–2019: A quasi-experimental and cost-effectiveness study

**DOI:** 10.1371/journal.pmed.1004212

**Published:** 2023-04-18

**Authors:** Justin S. White, Sanjay Basu, Scott Kaplan, Kristine A. Madsen, Sofia B. Villas-Boas, Dean Schillinger

**Affiliations:** 1 Philip R. Lee Institute for Health Policy Studies, University of California, San Francisco, California, United States of America; 2 Waymark Health, San Francisco, California, United States of America; 3 Department of Economics, United States Naval Academy, Annapolis, Maryland, United States of America; 4 School of Public Health, University of California, Berkeley, California, United States of America; 5 Department of Agricultural & Resource Economics, University of California, Berkeley, California, United States of America; 6 Center for Vulnerable Populations, Division of General Internal Medicine, San Francisco General Hospital/University of California San Francisco, San Francisco, California, United States of America

## Abstract

**Background:**

While a 2021 federal commission recommended that the United States government levy a sugar-sweetened beverage (SSB) tax to improve diabetes prevention and control efforts, evidence is limited regarding the longer-term impacts of SSB taxes on SSB purchases, health outcomes, costs, and cost-effectiveness. This study estimates the impact and cost-effectiveness of an SSB tax levied in Oakland, California.

**Methods and findings:**

An SSB tax ($0.01/oz) was implemented on July 1, 2017, in Oakland. The main sample of sales data included 11,627 beverage products, 316 stores, and 172,985,767 product-store-month observations. The main analysis, a longitudinal quasi-experimental difference-in-differences approach, compared changes in beverage purchases at stores in Oakland versus Richmond, California (a nontaxed comparator in the same market area) before and 30 months after tax implementation (through December 31, 2019). Additional estimates used synthetic control methods with comparator stores in Los Angeles, California. Estimates were inputted into a closed-cohort microsimulation model to estimate quality-adjusted life years (QALYs) and societal costs (in Oakland) from 6 SSB-associated disease outcomes. In the main analysis, SSB purchases declined by 26.8% (95% CI −39.0 to −14.7, *p* < 0.001) in Oakland after tax implementation, compared with Richmond. There were no detectable changes in purchases of untaxed beverages or sweet snacks or purchases in border areas surrounding cities. In the synthetic control analysis, declines in SSB purchases were similar to the main analysis (−22.4%, 95% CI −41.7% to −3.0%, *p* = 0.04). The estimated changes in SSB purchases, when translated into declines in consumption, would be expected to accrue QALYs (94 per 10,000 residents) and significant societal cost savings (>$100,000 per 10,000 residents) over 10 years, with greater gains over a lifetime horizon. Study limitations include a lack of SSB consumption data and use of sales data primarily from chain stores.

**Conclusions:**

An SSB tax levied in Oakland was associated with a substantial decline in volume of SSBs purchased, an association that was sustained more than 2 years after tax implementation. Our study suggests that SSB taxes are effective policy instruments for improving health and generating significant cost savings for society.

## Abstract 2

Justin White and co-workers report an evaluation of the 2017 sugar-sweetened beverage tax enacted in Oakland in California.

## Author summary

**Why was this study done?:**

- Several US cities have implemented a sugar-sweetened beverage (SSB) tax aimed at reducing SSB consumption. Yet, there remains relatively little evidence regarding the sustained effects of SSB taxes.
- Few prior studies of SSB taxes have accounted for the multiple ways consumers might compensate for a tax.
- Evidence regarding the cost-effectiveness of SSB taxation in the US has been based on projections of a national tax, rather than empirical estimates from local taxes.

**What did the researchers do and find?:**

- We applied difference-in-differences and synthetic control approaches comparing changes in SSB purchases before versus after the SSB tax in Oakland with changes in comparator areas.
- The excise tax was associated with a 26.8% reduction in SSB purchases in Oakland during the first 30 months after tax implementation, based on the difference-in-differences analysis. The results were similar using a synthetic control approach. We do not find evidence of cross-border shopping or caloric substitution resulting from implementation of the tax.
- We used the estimated effects of Oakland’s SSB tax as an input into a microsimulation model of the quality-adjusted life years (QALYs) and societal costs from 6 SSB-associated disease outcomes.
- The reduction in SSB purchases implied improvements in health outcomes (0.15% QALYs gained) and substantial cost savings (>$100,000 per 10,000 people) over a 10-year horizon, with larger gains over a lifetime horizon.

**What do these findings mean?:**

- Our findings suggest that local SSB taxes can improve dietary intake and result in meaningful improvements in health. They are also expected to generate cost savings.
- These results provide additional support for a recent recommendation to Congress from a federal diabetes commission to implement a national excise tax on SSBs.

## 1. Introduction

Sugar-sweetened beverages (SSBs) constitute a major, nonnutritional source of calories and added sugars in the US diet and are associated with obesity, type 2 diabetes, and overall mortality [1–3]. As of 2021, excise taxes had been levied on beverage distributors in 7 US localities and more than 35 countries to reduce the risk of diet-sensitive chronic disease and increase government revenue for health promotion [4,5].

SSB taxation has been considered by many to be among the most promising population-level interventions to prevent and control chronic disease [6]. Following implementation of Berkeley’s first-in-the-nation SSB tax in 2015, a growing number of studies have documented the benefits of US municipal-level taxes on consumers [7]. This body of literature has shown that SSB taxes increase retail SSB prices for consumers, leading to short-term reductions in purchase and intake of SSBs [8–11]. What remains unclear is whether SSB taxation produces sustained declines in SSB intake, and the extent to which consumers substitute SSBs for other foods or beverages or cross borders to shop in untaxed locations. In addition, the net effect of SSB taxes on health and costs when considering the health profiles of exposed populations has not been estimated [12–14]. A 2021 federal commission on diabetes recommended that the US government levy a federal SSB tax to improve diabetes prevention and control efforts [15]. This study aimed to provide critical and timely evidence on beverage purchasing patterns and health and cost impacts to inform such a policy.

On July 1, 2017, the city of Oakland, California implemented a $0.01/oz tax on the distribution of all beverages ≥12 ounces containing added sugar. We evaluated whether the SSB tax in Oakland, California was associated with reduced SSB purchases up to 30 months after tax implementation. Prior research has found that the Oakland tax increased retail prices of SSBs by approximately 14%, although evidence of changes in SSB purchasing and intake following the tax has been mixed [16–20]. We also assessed changes in purchases of untaxed beverages, cross-border purchases, compensatory food purchases, and subgroup differences by beverage category, beverage size, store type, and area-level household income. Finally, we performed a microsimulation to estimate the health and cost impact across major disease outcomes associated with SSB intake, ensuring that our results are informative to ongoing policymaking.

## 2. Methods

This study used retail sales data and difference-in-differences and synthetic control approaches to estimate the impact of the SSB tax on beverage purchases. A microsimulation model used the quasi-experimental estimates and a variety of survey data (described below) to implement a microsimulation model of the cost-effectiveness of the SSB tax. All analyses were prespecified (S1 Prospective Plan). The study follows Consolidated Health Economic Evaluation Reporting Standards (S1 Checklist).

### 2.1. Data

Retail sales data (described elsewhere [21]) were licensed from Information Resources Inc. (IRI), which obtains data from major US retailers. IRI establishes relationships with store chains and independent stores to share electronic data regarding all sales transactions at participating stores. Studies have found that sales in IRI retail scanner data align well with other sources of retail sales data [22]. Longitudinal sales data were provided in 4-week periods, hereafter monthly, for beverages purchased from January 1, 2015 (30 months before-tax in Oakland) through December 31, 2019 (30 months after-tax). Data were provided at the individual product level based on a unique universal product code (UPC) and contained monthly store-level volume and dollar sales of each beverage purchased (11,627 UPCs). Our data files contain the total sales value and volume by store for each beverage product and selected food products during a 4-week period.

### 2.2. Beverage classification

Beverages were classified as taxed or untaxed based on Oakland tax regulations and classified into 8 taxed and 15 untaxed beverage categories (see S1 Text). Nutrient and ingredient data were drawn from Label Insight, the then-largest publicly available branded food composition database in the US [23,24], and a hand-coding procedure from online searches (S1 Text). Beverages were also classified as individual- or family-sized (defined as ≥36 oz) [25].

### 2.3. Outcomes

The primary outcome was the average monthly change in before- versus after-tax beverage volume purchased in fluid ounces per store.

### 2.4. Statistical analyses

This study used a difference-in-differences approach to compare beverage purchases before versus after an SSB tax in Oakland (the intervention city) compared with those in Richmond (a noncontiguous comparator city 12 miles from Oakland with a similar sociodemographic and health profile [S1 Table]). Difference-in-differences is a common quasi-experimental technique used to evaluate health policies, including SSB taxes, when the exposure is not randomly assigned, because of its ability to restrict the scope of potential confounding from secular trends [11,26,27]. Secondarily, we conducted a synthetic control approach that constructed a “synthetic” Oakland by assigning weights to stores from our comparator location, Los Angeles (LA), using pretax SSB purchases and a set of location-specific prognostic factors [28]. Synthetic control methods take a data-driven approach to constructing a comparison group and may reduce susceptibility of estimates to unmeasured time-varying confounders [29].

#### 2.4.1. Main analysis of volume purchases

The main analysis, conducted at the UPC level, uses a difference-in-differences approach to assess changes in volume of SSBs purchased before versus after tax for Oakland compared with Richmond. By adjusting for the counterfactual change in outcomes as measured in the comparator city, differences-in-differences allows one to attribute any differential changes in Oakland to the tax. The primary independent variable in the difference-in-differences analysis is an indicator variable for whether the observation was recorded in Oakland after implementation of the tax. The difference-in-differences analysis also included fixed effects (i.e., indicators) for store, year-quarter, and UPC. The regressions therefore adjusted for measured or unmeasured confounders that were unchanged during the study period within store (e.g., store type), within the store’s neighborhood (e.g., racial/ethnic composition), within year-quarter (e.g., seasonality), or within UPC (e.g., package size, calories) [27,30]. The fixed effects account for any changes over time in sample composition of stores or UPCs. In particular, UPCs discontinued prior to the tax or introduced after the tax are not a source of variation used to identify (or bias) the difference-in-differences estimate. Standard errors were clustered by zip code to account for correlated outcomes over time. *P* values were corrected for multiple hypothesis testing using Anderson’s sharpened False Discovery Rate procedure [31]. See S1 Text for additional details.

Beverage substitution was also assessed with a difference-in-differences regression of purchases of untaxed beverages. It compares pre-post changes in purchases of untaxed beverages in Oakland while differencing out secular trends in purchases of untaxed beverages in comparator city Richmond. Cross-border shopping was assessed with a difference-in-differences regression of SSB purchases in the border areas within approximately 3 miles [32] of Oakland versus in equidistant border areas of Richmond. Compensatory purchases of sweet foods have been hypothesized to increase following an SSB tax as consumers search for cheaper sources of sugar [33–35]. Compensatory food purchases were assessed with a difference-in-differences regression of selected sweet snacks (packaged baked goods, 5,130 UPCs) in Oakland compared with Richmond. Subgroup analyses were performed by beverage category (listed in S1 Text), store type (convenience, pharmacy, supermarket/grocery store), beverage size (individual- versus family-sized), and income around the store’s location (store zip code with above- versus below-median proportion of households with <$35,000 income). All difference-in-differences analyses included fixed effects for store, year-quarter, and UPC.

#### 2.4.2. Secondary and sensitivity analyses of volume purchases

To examine dynamic treatment effects, we estimated an event study difference-in-differences regression of the time-varying association between the tax and SSB purchases [27,36]. The event study provided quarter-by-quarter estimated effects [37]. The significance of the before-tax coefficients provided a diagnostic of parallel pre-event outcome trends, an indicator of whether the identifying assumption of difference-in-differences estimation may be satisfied [37,38].

Finally, while selecting nearby Richmond as a comparison city accounted for any potential effects of SSB tax-related media on purchases, its proximity meant that SSB purchases in Richmond could have increased after the tax due to cross-border purchasing from Oakland into Richmond city proper or border areas. Therefore, we also conduct a secondary analysis that estimated a synthetic control model with a matched comparison group constructed from stores in LA, California. The synthetic control method algorithmically found the weighted average of LA stores that best matched the trend of before-tax values of the outcome and prognostic covariates in Oakland [28,29]. In this way, the method may be more robust to unmeasured time-varying confounders [28], although it relies on aggregated data by store. We considered as prognostic covariates several sociodemographic characteristics of the store zip code from the 2016 American Community Survey and the 2010 US census, including: population size (2010), median household income (2016), racial/ethnic composition (proportion non-Hispanic White, non-Hispanic Black, Hispanic, and Asian, 2010), proportion in poverty (household income <$10,000, 2016), proportion aged 18 to 24 (2010), and number of housing units (2010). Prognostic variables were selected due to their hypothesized relationship with SSB purchases, informed by prior literature [39–41]. Uncertainty estimates and statistical significance of the model were based on placebo treatment effects for all stores in the donor pool, as if each control store had been treated. A randomization inference approach was used to calculate whether or not the estimated effect of the actual treated unit is large relative to the distribution of placebo effects (i.e., the proportion of control units that have an estimated placebo effect as large as that of the treated unit) [28].

We conducted sensitivity analyses for the synthetic control estimates for specifications that included Richmond (as well as LA) stores in the donor pool of comparator stores, adjustment for average retail price of taxed products per ounce as an additional covariate (omitted from the main model because it is endogenous to price), a cross-validation procedure of the four quarters before the tax with the early pretax period acting as a training period [29] and restricting the sample to a set of products that were available for all time periods.

#### 2.4.3. Microsimulation model

We simulated the population of Oakland to determine the anticipated effect of the tax-induced decline in SSB intake on quality-adjusted life years (QALYs) and societal costs from SSB-associated disease outcomes. We used demographic data from the American Community Survey (2019) from Oakland’s Alameda County and assigned simulated persons before-tax SSB intake levels and disease risk factors/history (S6 Table) by repeatedly sampling from age-, sex-, race-, and ethnicity-matched participants in the National Health and Nutrition Examination Survey (NHANES, 2015 to 2018). Following our previously published simulation for modeling the impact of SSB intake changes on health and cost outcomes [42], we estimated 6 disease outcomes: obesity (body mass index [BMI] of ≥30 kg/m^2^) [43], coronary heart disease (angina, myocardial infarction, cardiac arrest, ischemic heart disease, or heart failure) [44], cerebrovascular accident (ischemic or hemorrhagic stroke) [45], type 2 diabetes mellitus (hemoglobin A1c of ≥6.5%, fasting plasma glucose of ≥126 mg/dL, or 2-hour oral glucose tolerance test result of ≥200 mg/d) [46], chronic kidney disease (estimated glomerular filtration rate of <90 mL/min [47], using the Chronic Kidney Disease-Epidemiology [CKD-EPI] equation) [48], and both dental caries and periodontal disease (tooth decay of the permanent teeth, periodontitis, or more advanced disease, based on clinical attachment loss and periodontal probing depth) [49]. We used prior estimates of the demographic-specific incidence rate and mortality associated with these outcomes (S6 Table) [50] and then reduced the incidence rates to reflect the relative risk reductions associated in each incidence rate and mortality from reduced SSB intake. Binomial random variables were drawn with probability equal to the incidence rate of each condition by demographics (S9 Table), and fatality rates calibrated to match the demographic mortality rate estimates (S10 Table). Without the SSB tax, the baseline levels of SSB consumption and associated outcome incidence were assumed. With the SSB tax, the levels of tax reduction were first assumed to equal the level of SSB consumption reduction, and the relative risk shown in the main text from meta-analytic estimates were used to reduce the incidence rate for each outcome (see S1 Text).

We first simulated reductions in SSB intake in Oakland based on the point estimates and 95% confidence intervals from our difference-in-differences estimates, assuming the percent reduction in purchasing equaled the percent reduction in consumption [51,52]. We repeatedly sampled from normal distributions constructed around the difference-in-differences point estimates and their 95% confidence intervals to construct uncertainty estimates around our simulated outcomes. We also simulated how much cross-border purchasing and substitution to other foods and untaxed beverages would have to occur to negate the tax effects (the “point of cost neutrality” in a threshold analysis [53]). The level of SSB consumption reduction was varied until we found the point which the healthcare cost savings from the tax was equal to the cost of the tax itself, estimating the tax cost as the increased price per ounce observed in the scanner data, multiplied by the level of consumption reflected in the posttax environment for each county. This inherently assumes that the tax itself produces a deadweight loss of market inefficiency that amounts to a societal cost [54,55]. We note the work of Alcott and colleagues [56] that indicates the deadweight loss itself can be challenging to identify for an SSB tax due to difficulty calculating how much the tax corrects for internalities (i.e., the costs that consumers impose on themselves).

For all health outcomes except obesity, we estimated the disutility of each outcome to calculate QALYs based on a survey-based assessment [57]. For obesity, we adopted a prior review of BMI-specific disutility [58], weighted by the BMI distribution in NHANES to assess quality-of-life losses beyond those related to the aforementioned outcomes (e.g., for obesity-related liver disease).

We obtained healthcare costs (inpatient, outpatient, and prescription drug spending) per patient from the Medical Expenditure Panel Survey (MEPS, 2018) and attendant surveys for other obesity complications (S6 Table). We included healthcare costs specific to the type of primary insurance coverage (commercial, Medicare, Medicaid/other public, or uninsured; S6 Table). We adjusted costs for inflation to January 2021 US dollars using the Consumer Price Index [59]. We then computed cost and disutility from both a healthcare perspective (including healthcare costs) and societal perspective (including both healthcare costs and the costs of the tax) on both 10-year and lifetime horizons and discounted at a standard 3% annual rate (S1 Checklist) [60,61]. We performed uncertainty analyses by repeated Monte Carlo sampling from the distributions of each input parameter, by specifically constructing a log-normal distribution based on the mean input parameter estimate and the standard deviations around those means for each input parameter (see S6 Table). After rerunning the model 10,000 times, the number of iterations that resulted in stability of the model results to rounding error, we tabulated the 95% credible intervals around the distribution of each outcome for each city’s population to provide a sense of the uncertainty bounds around the modeling results.

The research was determined not to meet the criteria for human participant research of the institutional review board at the University of California San Francisco.

## 3. Results

### 3.1. Store and beverage sample

The main analytic sample included 172,985,767 product-store-month observations from 316 stores, 174 within the city limits of Oakland and Richmond, and 142 within their border areas. Mean store volume purchased of all beverages and SSBs in 2016 (the year before Oakland’s tax) were somewhat higher in Richmond than Oakland, although the percent of volume purchases that were SSBs was similar across locations (Table 1).

**Table 1:** Descriptive statistics of aggregated beverage sales, 2016.

			Average beverage purchases per store in city limits, 2016					
	Number of stores		All beverages			SSBs		
	City limits	Border areas	Oz, in mil	$, in mil		Oz, in mil	$, in mil	% SSBs of total Oz^a^
Oakland	42	98	5.71	0.73		1.90	0.21	33.33
Mass merchandise stores	1	28						
Convenience stores	11	6						
Pharmacies	19	42						
Supermarkets	11	22						
Richmond	9	24	7.82	0.84		2.56	0.29	32.73
Mass merchandise stores	3	5						
Convenience stores	2	1						
Pharmacies	3	11						
Supermarkets	1	7						
Los Angeles^b^	223	83	9.25	1.09		3.38	0.30	36.55
Mass merchandise stores	32	10						
Convenience stores	26	9						
Pharmacies	110	43						
Supermarkets	61	21						
Synthetic Oakland^c^	128					1.81	0.21	
Total (main analysis)^d^	174	142	6.0	0.75		2.01	0.22	33.22
Total (all stores)	403	225	8.6	1.03		3.12	0.29	35.92

^a^SSB volume purchases as a percentage of total beverage volume purchases.

^b^Los Angeles is included in the synthetic control analysis only.

^c^Weighted average of sales in Los Angeles stores that best matches sales in Oakland based on the base model from the synthetic control analysis.

^d^Includes Oakland and Richmond only, the cities included in the difference-in-differences analysis.

Of the 11,627 beverage UPCs, we were able to classify the tax status of 10,219 UPCs (87.9%) and the beverage category of 10,942 UPCs (94.1%). Classified UPCs included 3,451 (33.8%) SSBs and 6,768 (66.2%) untaxed beverages. We were able to ascertain tax status and beverage category for products that constituted 96.9% of sales volume in the data in Oakland and 97.1% in Richmond.

### 3.2. Main analysis of volume purchases

SSB purchases declined by 26.8% (95% CI −39.0 to −14.7, *p* < 0.001) in the 30 months after tax implementation in Oakland compared with Richmond, an average monthly change of −45,220 oz per store (95% CI −65,667 to −24,773, *p* < 0.001) according to the difference-in-differences estimate (Table 2). There was no significant change in untaxed beverage purchases, cross-border SSB purchases compared with Richmond border areas, or compensatory purchases of sweet snacks (Table 2).

**Table 2:** Difference-in-differences estimates of change in volume purchased per store in Oakland.

	No. UPCs	Adjusted Difference-in-Differences Model		Adjusted % Change^c^
		Estimate (95% CI), in 100s oz^a^	*P* value^b^	
All beverages				
SSBs	3,451	−452.20 (−656.67 to −247.73)	<0.001	−26.83
Untaxed beverages	6,768	−154.99 (−584.86 to 274.88)	0.21	−4.91
SSBs by beverage category				
Soda	1,477	−86.64 (−122.44 to −50.81)	<0.001	−23.13
Fruit drinks	473	−83.83 (−141.14 to −26.54)	0.006	−30.43
Sports drinks	263	−214.15 (−318.83 to −109.49)	<0.001	−42.43
Energy drinks	93	−3.44 (−10.19 to 3.32)	0.16	−49.17
Coffee	320	−0.75 (−6.40 to 4.93)	0.31	−1.2
Tea	504	−74.84 (−117.13 to −32.56)	0.003	−24.43
Flavored water	103	2.26 (1.24 to 3.29)	<0.001	99.04
SSBs by store type				
Convenience stores	3,451	−51.31 (−110.91 to 8.28)	0.06	−10.85
Pharmacies	3,451	−17.72 (−70.74 to 3.531)	0.22	−4.28
Supermarkets	3,451	−1,837.67 (−2,298.94 to −1,376.40)	<0.001	−38.41
SSBs by beverage size^d^				
Individual	2,083	−262.96 (−382.21 to −143.72)	<0.001	−28.34
Family	1,368	−190.52 (−289.36 to −91.68)	<0.001	−25.32
SSBs by zip-code level income^e^				
Stores in lower income area	3,451	−309.02 (−395.33 to −222.72)	0.003	−24.95
Stores in higher income area	3,451	−570.12 (−926.24 to −214.00)	0.003	−26.49
SSBs in border areas^f^	3,810	−30.55 (−479.61 to 418.51)	0.33	−1.25
Sweet snacks^g^	5,130	0.38 (−5.10 to 5.86)	0.33	1.88

^a^The adjusted difference-in-differences estimates is the mean change in volume purchased per store, in 100s of fluid ounces, from regression models that include store fixed effects, UPC fixed effects, and year-quarter fixed effects. For sweet snacks, volume is measured in 100s of ounces.

^b^*P* values are corrected for multiple hypothesis testing using Anderson’s sharpened False Discovery Rate procedure [31].

^c^The percent change was calculated by dividing the difference-in-difference estimate by the average before-tax volume in the intervention city. The numerator represents the change in volume sales in the after-tax period compared with the before-tax period controlling for secular trends using Richmond as a comparator.

^d^Beverages are individual-sized if 36 fl oz or less and family-sized if more than 36 fl oz.

^e^Income was based on zip code-level data from 2016 5-year American Community Survey estimates. Zip codes with a below-median proportion of residents living under $35,000 were considered a lower-income area, and zip codes with an above-median proportion of residents living under $35,000 were considered a higher-income area.

^f^This compares border zip codes around Oakland to the border zip codes around Richmond.

^g^This includes all products within the retail categories of “doughnuts” and “cookies.”

Following the tax, SSB purchases in Oakland declined for several beverage categories, including sweetened soda (−23.1%; −8,664 oz per store, 95% CI −12,244 to −5,081, *p* < 0.001), fruit drinks (−30.4%; −8,383, 95% CI −14,114 to −2,654, *p* = 0.006), sports drinks (−42.4%; −21,415, 95% CI −31,883 to −10,949, *p* < 0.001), and sweetened teas (−24.4%; −7,484, 95% CI −11,713 to −3,256, *p* < 0.003). SSB purchases declined by 38.4% in supermarkets (−183,767 oz per store, 95% CI −229,894 to −137,640, *p* < 0.001) but did not significantly change in convenience stores or pharmacies. We found similar declines in SSB purchases for individual-sized (−28.3%; −26,296 oz per store, 95% CI −38,221 to −14,372, *p* < 0.001) and family-sized (−25.3%; −19,052, 95% CI −28,936 to −9,168, *p* < 0.001) products, and for stores in lower-income (−25.0%; −30,902, 95% CI −39,533 to −22,272, *p* = 0.003) and higher-income (−26.5%; −57,012, 95% CI −92,624 to −21,400, *p* = 0.003) areas.

### 3.3. Secondary analyses of volume purchases

#### 3.3.1. Change in volume purchases over time (event study)

**Fig 1:**
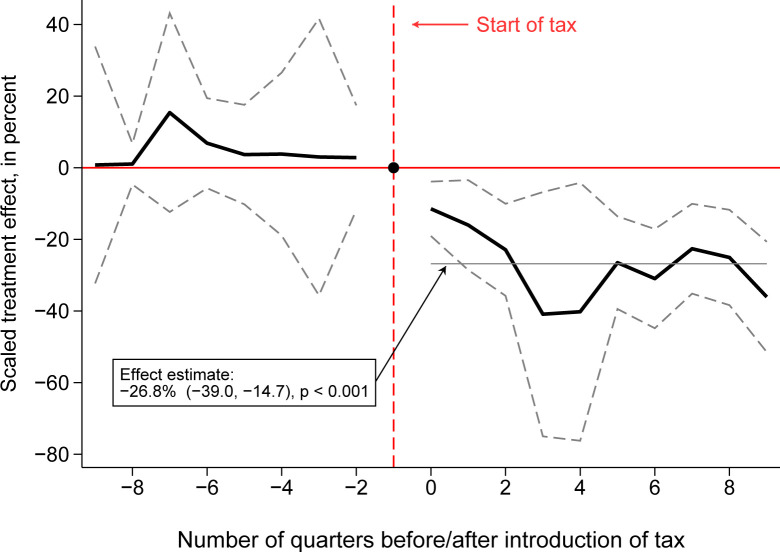
Time-varying association between the Oakland tax and volume sales of SSBs (plots from event study difference-in-differences models). Note: This plot shows the change in average monthly ounces sold per product in a store, based on an event study difference-in-differences regression. Quarterly estimates are relative to the quarter before the tax (quarter −1, red dotted line). Gray dotted lines are 95% confidence intervals from robust standard errors clustered by zip code.

The event study estimates in the pretax period are not statistically different from zero, suggesting that volume sales in the intervention and comparator cities did not change differentially prior to tax implementation (Fig 1). This finding supports the parallel trends condition for identification of the difference-in-differences model. The magnitude of the association between the tax and purchases grew in Oakland during the first 2 year-quarters after-tax, remaining stable thereafter.

#### 3.3.2. Synthetic control estimates of volume purchases

**Fig 2:**
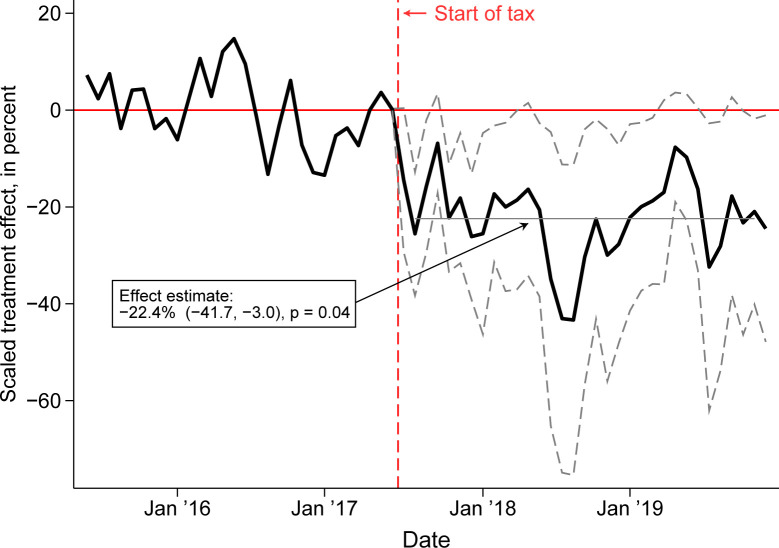
Synthetic control estimates of the association between the Oakland tax and the volume of SSB purchases. Note: This figure displays the proportionate change in the volume of SSB purchases in Oakland compared with a synthetic control group composed of a data-driven weighted average of stores in LA. Gray dotted lines are 95% confidence intervals.

SSB purchases in Oakland declined by 22.4% (95% CI −41.7% to −3.0%, *p* = 0.04) following tax implementation compared with a synthetic control group of LA stores (Fig 2, Table 3). In sensitivity analyses, estimates were similar in all specifications except for a muted response when using a set of products available in all time periods (−15.1%) (Table 3). Restricting the data to a consistent set of products provides a stable comparison group, but it could deliver biased estimates if the rate of products entering the market changes over time.

In synthetic control analyses, cross-border SSB purchases did not change in Oakland border areas relative to LA border areas (Table 3).

**Table 3:** Sensitivity analyses of the synthetic control estimates of the association between the Oakland tax and the volume of SSB purchases.

	Coef. (95% CI)
*Panel A*. *SSB purchases in city limits*	
Base model: LA-only synthetic control group	−0.224 (−0.417 to −0.030)
*Panel B*. *Sensitivity analyses*	
LA and Richmond synthetic control group	−0.233 (−0.428 to −0.038)
Adjust for retail price per oz^a^	−0.224 (−0.410 to −0.039)
Cross-validation^b^	−0.231 (−0.429 to −0.032)
Panel of products^c^	−0.151 (−0.333 to 0.032)
*Panel C*. *Cross-border SSB purchases in border areas*	
Oakland border area	0.041 (−0.062 to 0.145)

Note: This table displays the proportionate change in the volume of SSB purchases in Oakland compared with a synthetic control group composed of a data-driven weighted average of stores in LA (Panel A) or compared with various alternative model specifications (Panel B) and a proportionate change in volume of SSB purchases in Oakland border areas with a synthetic control group composed of stores in LA’s border area (Panel C). Each row represents a different synthetic control model. Coefficient is calculated as the mean estimate of the association between the tax and volume sales during the posttax period.

Prediction error as great as the intervention city or cities.

^a^Adjusts for average retail price of taxed products per ounce as an additional covariate (omitted from the main model because it is endogenous to—affected by—the tax.

^b^Uses cross-validation of the later pretax period with the early pretax period acting as a training period.

^c^Restricts the sample to UPCs that were available for all time periods.

### 3.4. Microsimulation estimates

Summary statistics of model input parameters are provided in S6 Table. The simulated Oakland population, based on the ACS (2019), had a median age of 36.9 years old, with 66% of the population aged 18 to 64; 52% male; and 29% White, 24% Black, 14% Asian, and 27% Hispanic (including all races under Hispanic ethnicity; S6 Table).

The simulated population, based on NHANES populations sampled and weighted to reflect the American Community Survey demographics of Alameda County, had a 17.4% prevalence of obesity, 5.2% prevalence of coronary heart disease, 3.0% prevalence of cerebrovascular accident, 7.9% prevalence of diabetes, 11.3% prevalence of chronic kidney disease, and 82.1% prevalence of dental disease (S6 Table); the first 4 statistics were possible to validate against the California Health Interview Survey (CHIS) and were found to be within 0.1% absolute percentage points of estimates from CHIS for Alameda County (2019) [62].

The simulated Oakland population, in the context of the SSB tax, was estimated to reduce net SSB intake by 26.8%, or 1.33 oz per person per day. The reduced intake was estimated to accrue 94.0 QALYs per 10,000 population over 10 years, and 967.6 QALYs over a lifetime (Table 4). From a societal perspective, the net tax impact per 10,000 people would save an estimated $102,154 over 10 years ($1.02 per person per year) and $1,251,817 over a lifetime ($2.81 per person per year). The long-term cost savings would be fully negated if the 26.8% reduction in SSB purchases resulted in a reduction in actual intake of only 0.3% (e.g., due to cross-border purchases or dietary substitution).

**Table 4:** Model estimates of SSB tax impact on QALYs and costs in Oakland, per 10,000 people.

Tax-attributable change in outcomes	10-year horizon	Lifetime horizon
Costs (negative costs = net savings)		
Healthcare	−$106,108 (−$10,739 to −$251,453)	−$1,269,770 (−$96,159 to −$3,163,781)
Societal (Healthcare + tax)	−$102,154 (−$6,785 to −$247,589)	−$1,251,817 (−$78,207 to −$3,145,828)
QALYs	94.0 (5.2 to 235.8)	967.6 (70.4 to 2,446.0)

Note: Model estimates were integrated on both a 10-year time horizon and a lifetime horizon (i.e., a life-course perspective), with the healthcare perspective incorporating only costs of treating health outcomes (specific to insurance type; see S6 Table) and the societal perspective additionally including the cost of the tax (both the portion absorbed by distributors and the portion paid by consumers). 95% credible intervals are in parentheses.

QALY, quality-adjusted life year; SSB, sugar-sweetened beverage.

## 4. Discussion

This study used rigorous quasi-experimental methods to examine the association between the Oakland SSB tax and sustained changes in volume purchases up to 30 months after-tax. Following the tax, SSB purchases declined by 26.8% in Oakland relative to a comparator city in the same market. This effect size, when combined with a retail price increase of 14% [18], corresponds to a price elasticity estimate of −1.9, in line with meta-analytic evidence from other studies (mean −1.6, 95% CI −2.1 to −1.1) [11]. The estimated decline in SSBs from the difference-in-differences analysis was similar when using a matched sample of LA stores in the synthetic control analysis (22.4%). These SSB reductions would be expected to deliver significant health improvements over 10-year and lifetime horizons, respectively. The QALYs gained would represent an increase of approximately 0.15% over 10 years, growing to 0.35% over a lifetime. The SSB reductions were also projected to save $1,088 per QALY saved (>$100,000 per 10,000 people) over a 10-year horizon and $1,294 per QALY saved (>$1M per 10,000 people) over a lifetime horizon. This adds to the evidence base that SSB taxes are projected to be highly cost-effective or even cost-saving [12–14,63].

To give a sense of how our cost-effectiveness results compare to other public health interventions, prior studies estimate that a clinical child obesity intervention would have a cost of $774 per QALY saved; eliminating the tax subsidy for television advertising would generate cost savings of $0.10 per QALY saved; a $1 cigarette tax (20% to 40% increase in price across states compared with approximately 15% associated with Oakland’s SSB tax [18]) plus anti-smoking media campaign would generate cost savings of $573,346 to $797,701 per QALY saved; smoke-free workplace policies have a cost of $726 per QALY saved; and traffic-related air pollution measures such as low-emission zones have a cost of $3,023 (£2,465) per QALY saved [64–67]. Thus, the SSB tax is estimated to be less cost-effective than the cigarette tax plus media campaign but more cost-effective than the smoke-free workplace policy and air pollution measures.

We investigated several potential ways that consumers may have compensated for the tax—substituting to untaxed beverages or high-sugar foods or shopping in untaxed neighboring jurisdictions. We did not find significant increases in purchases of untaxed beverages, neither in aggregate nor for different categories of untaxed beverages. Furthermore, we did not observe offsetting caloric compensation in Oakland, adding to prior evidence finding little or no caloric compensation following SSB taxes [33–35]. In addition, we did not find significant cross-border shopping, a point we return to in the next paragraph.

Prior studies have found that Oakland’s tax increased SSB prices by roughly 14% to 15% [16–20] and reported a more muted after-tax decline in SSB sales. One study found no decline in SSB purchases 1 year after-tax, although it included only 34 households in Oakland [20]. A pair of studies found declines in retail sales in Oakland of 14% to 18% 1 and 2 years posttax, compared with Sacramento, which was offset by roughly half due to cross-border shopping [19,68]. In contrast, we found greater reductions in SSB purchasing and no significant cross-border shopping across several model specifications. We speculate that estimates may, in part, be sensitive to estimation approach, data set, and the choice of comparator. We assessed Richmond’s validity by conducting diagnostic tests of parallel before-tax trends and found Richmond’s city limits and border area to be a well-matched comparator to Oakland’s city limits and border areas. We were able to adjust for UPC and store characteristics, removing many potential confounders. Our findings were further supported in our complementary analysis that used a synthetic control; the use of the alternative, data-driven control group yielded a similar estimate of the tax’s effect and no significant changes in cross-border shopping. That said, even if our estimated tax effects were offset by half due to cross-border shopping, the effect size would remain substantial.

We observed heterogeneity in the response to the SSB tax, with SSB purchases declining more for several beverage categories (e.g., soda and sports drinks) than in others (sweetened coffee and flavored water). SSB purchases also declined most in supermarkets, where SSB prices in Oakland increased somewhat more after-tax than in other stores [16,18]. This finding is particularly important given that two-thirds of calories in the US diet come from grocery stores [69].

Insofar as recent policy recommendations have included scaling SSB taxation to the state or federal levels, the projected improvements in quality of life and cost savings we find are particularly relevant. While our findings are not necessarily generalizable to all US regions, studies of other US cities have found similar reductions in SSB purchases [25,33,70]. This suggests that our health and cost predictions may approximate the benefits that could accrue through a nationwide SSB tax. An SSB tax at the state or federal levels would be expected to provide fewer opportunities for cross-border shopping than a municipal tax, as most consumers would face high time and travel costs by trying to avoid the tax. Thus, our estimates contribute to understanding the benefits of state or federal taxation, regardless of whether the extent of cross-border shopping approximates our estimate or higher estimates from the literature.

### 4.1. Limitations

This study has several limitations. First, the tax status and beverage category could not be determined for a small proportion of products (constituting 3% of sales volume). However, any resultant bias was likely small, as non-ascertainment did not differ across cities. Second, our study relied on beverage purchases rather than consumption. Retail purchases closely correlate with consumption, although this correlation has been studied at the household, not individual level [51,52]. Survey-based measures of SSB intake may be subject to reporting bias [71] and are costly and challenging to collect across an entire population. We also found that the estimated decline in SSB purchases was 100-fold greater than the consumption decline needed for the tax to be cost-neutral, according to our sensitivity analysis. Third, because of the distance between Richmond to Oakland, cross-border shopping from Oakland to Richmond is possible and could lead to overestimation of tax effects in the difference-in-differences regressions. However, we failed to observe a crude increase in SSB purchasing in Richmond (S2 Table) or significant increases in the more proximate border areas of intervention cities, making this unlikely. This concern, in part, motivated the synthetic control analyses with an LA-only synthetic control group to avoid this potential bias; it too revealed no significant changes in cross-border shopping compared with LA border areas (Table 3). Fourth, the IRI data primarily reflect purchases in chain stores; our results may not apply to smaller and independent stores, such as corner liquor or convenience stores. In Philadelphia, independent stores experienced a 38% decline in SSB purchases following its SSB tax, which fell at the high end of estimates for SSB declines in large chains [33]. Insofar as independent stores are more prevalent in low-income neighborhoods, our estimates may understate differences in tax effects by area-level income. The fact that we observed similar reductions in higher- versus lower-income neighborhoods suggests that the SSB tax had substantial impact across the neighborhood income spectrum. Fifth, the availability of 2 cities only for the difference-in-differences analysis did not allow for clustering standard errors by city; rather, we cluster standard errors by zip code that may understate the degree of uncertainty in our estimates. Sixth, the microsimulation omitted any health or economic benefits of public investments using SSB tax revenues and did not estimate costs due to lost productivity and work absenteeism because we lacked data on which individuals purchased these products and their associated work histories. As such, the net benefits of SSB taxation we derived likely represent an underestimate [4,42,72]. Further research might also consider the cost-effectiveness of SSB taxation for key population subgroups, including differences by race/ethnicity, income, and chronic disease risk. A further limitation of our modeling analysis is that we cannot incorporate into the societal cost estimates the potential costs of taxation in terms of declining revenue to SSB companies (and associated surplus accumulation, or employment or economic contractions). However, such revenue effects remain debated as SSB company literature suggests that these companies rapidly pivot to new product entrances and alternatives such as zero-calorie beverages when SSB consumption declines [73].

## 5. Conclusions

Recent policy debates have focused on the potential role that SSB taxation can play to advance population health, and the issue has become contentious. Several states have bowed to pressure from the American Beverage Association to pass legislation that preempts new local SSB taxes [74,75]. California’s preemption law went into effect on June 28, 2018. As a result, public health advocates are now arguing for state-level taxation. A 2021 federal commission recommended that the US government implement an excise tax on SSBs as a strategy to control the diabetes epidemic, yet opponents often point to insufficient data [76].

We believe our study provides important new information to inform this public debate. Using retail sales data and modern natural experiments research designs, we observed a sustained, net reduction in the volume of SSBs purchased following tax implementation in Oakland. We estimate that this tax-attributable decline in SSB purchases translates into meaningful improvements in quality of life and significant societal cost savings. These results suggest that SSB taxation is an important policy lever to reduce diet-sensitive chronic disease risk and supports recommendations to scale to state or federal levels.

## Supporting information

S1 Checklist CHEERS checklist. (DOCX)

S1 Text Supplemental methods. (PDF)

S1 Prospective Plan Prospective plan. (PDF)

S1 Table Sociodemographic characteristics by city. (PDF)

S2 Table Volume sales per store before and after the Oakland tax in a panel of stores and products in Oakland and Richmond (a comparator). (PDF)

S3 Table Difference-in-differences estimates of change in volume sales of untaxed beverages in Oakland compared with Richmond. (PDF)

S4 Table Test of parallel pretrends in volume sales between Oakland and Richmond in event study difference-in-differences model. (PDF)

S5 Table Synthetic control estimates of the association between the Oakland tax and SSB volume sales in the Oakland border area. (PDF)

S6 Table Summary statistics on model input data. (PDF)

S7 Table Population-weighted estimates from the National Health and Nutrition Examination Study (2015–2018), sampled to represent the population of Alameda County. (PDF)

S8 Table Estimates of outcome event costs, $US2021 from the Medical Expenditure Panel Survey (2018). (PDF)

S9 Table Incidence rates for simulated outcome events per Global Burden of Disease estimates (2019). (PDF)

S10 Table Mortality rates for simulated outcome events per Global Burden of Disease estimates (2019). (PDF)

S1 Fig Average monthly ounces per product-store sold of SSBs and untaxed beverages. (PDF)

S2 Fig Time-varying association between the Oakland tax and volume sales of untaxed beverages (plots from event study difference-in-differences models). (PDF)

S3 Fig Time-varying association between the Oakland tax and volume sales of SSBs in the border areas of Oakland relative to the border areas of Richmond (plots from event study difference-in-differences models). (PDF)

S4 Fig Time-varying association between the Oakland tax and volume sales of SSBs by beverage category (plots from event study difference-in-differences models). (PDF)

S5 Fig Time-varying association between the Oakland tax and volume sales of SSBs by store type (plots from event study difference-in-differences models). (PDF)

S6 Fig Time-varying association between the Oakland tax and volume sales of SSBs by beverage size (event study plots from difference-in-differences models). (PDF)

S7 Fig Time-varying association between the Oakland tax and volume sales of SSBs by store area income (plots from event study difference-in-differences models). (PDF)

S8 Fig Time-varying association between the Oakland tax and volume sales of sweet snacks (plots from event study difference-in-differences models). (PDF)

S9 Fig Time-varying association between the Oakland tax and volume sales of SSBs compared with LA (event study plots from difference-in-differences models). (PDF)

S10 Fig Net present value of accrued disease-specific costs over time. (PDF)

## Abbreviations

BMI: body mass index
CHIS: California Health Interview Survey
CKD-EPI: Chronic Kidney Disease-Epidemiology
IRI: Information Resources Inc
MEPS: Medical Expenditure Panel Survey
NHANES: National Health and Nutrition Examination Survey
QALY: quality-adjusted life year
SSB: sugar-sweetened beverage
UPC: universal product code
