# Supplementary material for: Evaluation of the sugar-sweetened beverage tax in Oakland, United States, 2015–2019: A quasi-experimental and cost-effectiveness study
Source: PLoS Med. 2023 Apr 18;20(4):e1004212. doi: 10.1371/journal.pmed.1004212 (PMC10112812; doi:10.1371/journal.pmed.1004212)
Supplement: S1 Text — (PDF) [file pmed.1004212.s002.pdf]

S1 Text. Supplemental methods

1. Beverage Classification

The Oakland excise tax applies to the distribution of any beverage that contains one or more caloric sweeteners that has been added and that contains ≥25 kilocalories per 12 fluid ounces. The tax does not apply to alcoholic beverages, milk products, baby formula, beverages for medical use, 100% fruit juice, or beverages sweetened only with artificial sweeteners (e.g., diet soda).

SSB products were classified into eight beverage categories: soda, fruit drink, sports drink, energy drink, coffee, tea, flavored water, and other. Untaxed beverage products were classified into 15 beverage categories: fruit drinks, sports drinks, energy drinks, coffee, tea, flavored water, club soda/tonic water, bottled water, 100% fruit juice, diet soda, milk, milk alternatives, and other untaxed products.

Because IRI cannot reveal the specific store associated with each sales record, the tax status and beverage category associated with private-label beverages are typically unknown.

We relied principally on data from Label Insight to classify the SSB status of a product and to classify individual products into beverage categories. We supplemented these data with a hand-coding procedure, in which we performed an online search for each UPC in the data set and abstracted information on total calories, total sugar, added sugar per serving, serving size, and artificial sweeteners from the ingredients list and nutrition facts panel.

2. Difference-in-Differences Model

Model Description

The event study plots from the difference-in-differences estimator are shown in Figure 1 in the main text and in **S2 Figure** to **S7 Figure**.

We considered including Los Angeles as an additional comparator city in the difference-in-differences models. However, we found that Los Angeles failed to match the pre-tax outcome trends of the intervention city, violating the parallel trends assumption for difference-in-differences models for each intervention city (**S8 Figure**).

All difference-in-differences regressions included fixed effects (i.e., indicators) for store, year-quarter, and UPC. The regressions therefore adjusted for measured or unmeasured confounders that were unchanged during the study period within store (e.g., store type, ownership, location) within the store's neighborhood (e.g., racial/ethnic composition, competition, local policy environment), within year-quarter (e.g., seasonality), or within UPC (e.g., package size, ingredients, and calories) [1,2]. The fixed effects also accounted for any changes over time in sample composition of stores or UPCs. Standard errors were clustered by zip code to account for correlated outcomes over time. *P*-values were corrected for multiple hypothesis testing using Anderson's sharpened False Discovery Rate procedure [3].

As part of the subgroup analyses, we assessed heterogeneous policy effects by area-level income. Analyses indicated that the mean volume of SSBs sold per store per month was highest in areas with larger proportions of households with per-capita incomes of $25,000-35,000 annually, and areas with larger proportions of households in this income range appeared to respond more to the tax in terms of reduced purchases.

3. Synthetic Control Model

Model Description

Synthetic control methods overcome two key limitations of standard DD analysis: the need to have a very similar control population to compare against the taxed population (noting the fact that intervention cities may be socially/culturally unique), and the inability to account for time-varying unobserved confounders (such as changes in culture, or migration) during the post-tax period. Synthetic control methods match pre-tax covariates and outcomes between taxed and untaxed units by weighting each of the possible control units to create a better match for the

taxed units during the pre-tax period. Specifically, for each outcome Y_{it} for store i in quarter t, and X_{it} observed store-level covariates, the method chooses weights w_J to minimize the distance $(Y_{it}, X_{it}) - \sum_J w_j(Y_{Jt}, X_{Jt})$, or the difference in observed outcomes between the taxed and weighted untaxed units J before the tax [4,5].

We aggregated data to the store level for the analysis and in our main synthetic control model used all stores in Los Angeles as the donor pool of potential control units. This avoids any contamination in the donor pool due to cross-border shopping. The synthetic control method required a balanced panel of stores, with store data available in all months, and we therefore restricted the sample to stores that were available throughout the study period.

As potential covariates, we gathered sociodemographic characteristics of the store zip code from the 2016 American Community Survey and the 2010 US census, including population size (2010), median household income (2016), racial/ethnic composition (proportion non-Hispanic White, non-Hispanic Black, Hispanic, and Asian, 2010), proportion in poverty (household income <$10,000K, 2016), proportion aged 18 to 64 (2010), and number of housing units (2010). Following standard methodology, we tested a variety of covariate sets and pre-tax outcomes to find the model specification that minimized the mean square prediction error (i.e., provided the closest match to the treated units) during the pre-policy period. Uncertainty estimates and statistical significance of the model were based on placebo treatment effects for all stores in the donor pool, as if each control store had been treated. A randomization inference approach was used to calculate whether or not the estimated effect of the actual treated unit is large relative to the distribution of placebo effects (i.e., the proportion of control units that have an estimated placebo effect as large as that of the treated unit) [5]. We constructed confidence intervals on the basis of the *p*-values from the randomization inference approach [6].

We conducted several sensitivity checks: 1) using a donor pool of comparator stores in LA and Richmond, 2) adjusting for average retail price of taxed products per ounce as an additional

covariate (omitted from the main model because it is endogenous to—affected by—the tax), 3) using cross-validation of the later pre-tax period (the four quarters before the tax) with the early pre-tax period acting as a training period [4,7], and 4) restricting the sample to a panel of UPCs that were available for all time periods.

We further use our synthetic control approach to assess cross-border shopping, constructing the synthetic control from a donor pool of stores in the border areas of Los Angeles and Richmond. We conduct the same sensitivity checks for the cross-border shopping models as for the main models described above (**S5 Table**).

Results

Volume sales of SSBs in Oakland declined by 22.4% (95% CI -41.7% to -3.0%) following implementation of the tax, according to our base synthetic control model (Figure 2, Table 3 in main text). This is similar to our main difference-in-differences estimate for Oakland. There were 7 stores that contributed 1% or more to the synthetic control, with the largest weights being 31.7%, 25.8%, and 7.2%.

The synthetic control estimate was robust to a variety of sensitivity checks, although it was more muted under the model specification with a panel of stores and UPCs (-15.1% 95% CI -33.3% to 3.2%) (Table 3 in main text).

Results from the synthetic control models of cross-border shopping are shown in **S5 Table**. In the border areas of Oakland, the point estimates from the various sensitivity analyses indicated increased sales in border areas of 2.0% to 9.1%, implying a modest amount of cross-border shopping, although none of the estimates were statistically significant.

4. Microsimulation Model

Model Input Data

Following our previously-published simulation of the modeled impact of SSB intake changes on health and cost outcomes [8], we estimated six disease outcomes, based on our prior work and the robustness of prior evidence for their association with SSB intake: obesity (body mass index [BMI] of ≥30 kg/m2) [9], coronary heart disease (angina, myocardial infarction, cardiac arrest, ischemic heart disease, or heart failure) [10], cerebrovascular accident (ischemic or hemorrhagic stroke) [11], type 2 diabetes mellitus (hemoglobin A1c of ≥6.5%, fasting plasma glucose of ≥126 mg/dL, or two-hour oral glucose tolerance test result of ≥200 mg/d) [12], chronic kidney disease (estimated glomerular filtration rate of <90 mL/min [13], using the Chronic Kidney Disease-Epidemiology [CKD-EPI] equation) [14], and both dental caries and periodontal disease (tooth decay of the permanent teeth, periodontitis, or more advanced disease, based on clinical attachment loss and periodontal probing depth) [15]. We used prior estimates of the demographic-specific incidence rate and mortality associated with these outcomes (**S6 Table**),[16] then reduced the incidence rates to reflect reduced SSB intake.

The input data for the microsimulation model is summarized in **S6 Table**.

Model Description

The microsimulation modeling was organized into four steps, with open-source code available online to facilitate replication and extension, at: https://github.com/sanjaybasu/SSBtax. The CHEERS checklist for the model is provided in **S1 Checklist** [17].

The first step in the modeling utilized the R nhanesA package to download, parse and weight the 2015-2018 waves of NHANES. Data were missing for 9.6% of observations, and were imputed using multiple imputation with chained equations using the mice package for weighted predictive mean matching [18]. Nested and dependent questions were accounted for in the data parsing, and both of two 24-hour dietary recalls were used with macros applying a Box-Cox transformation and Monte Carlo sampling to estimate usual dietary intake for each sampled individual’s recalls (see **S7 Table**) [19].

The second step in the modeling used the tidycensus package to retrieve, parse, and weight the 2019 wave of ACS. Data were extracted at the county level for Alameda County, using Census tables B01001A through B01001G to extract age, sex, and race/ethnicity distributions by county. Missing data were present for 1.9% of observations, and were not imputed. The simulated population of each county was then constructed by simulating the age, sex, and race/ethnicity of individuals from the ACS data and repeated Monte Carlo sampling with replacement from their corresponding age, sex, and race/ethnicity-specific peers in NHANES to assign those individuals values for SSB consumption, prevalence co-morbidities and disease history, and insurance status (**S7 Table**).

The third step in the modeling used the MEPS package to extract the cost distribution by insurance type around each of the simulated outcomes from the 2018 wave of MEPS. The extraction used event data files h178assp through h178hssp linked to medical, pharmacy, and dental claims, then to condition files to compute weighted estimates by event types in allowed amount costs (**S8 Table**). Missing data were present for 8.1% of observations and were not imputed.

The fourth step in the modeling was simulating the outcomes and associated QALYs and costs with and without the SSB tax in place. Binomial random variables were drawn with probability equal to the incidence rate of each condition by demographics (**S9 Table**), and fatality rates calibrated to match the demographic mortality rate estimates (**S10 Table**). Without the SSB tax, the baseline levels of SSB consumption and associated outcome incidence were assumed. With the SSB tax, the levels of tax reduction were first assumed to equal the level of SSB consumption reduction, and the relative risk shown in the main text from meta-analytic estimates were used to reduce the incidence rate for each outcome. The level of SSB consumption reduction was then varied until we found the point which the healthcare cost savings from the tax was equal to the cost of the tax itself, estimating the tax cost as the increased price per ounce observed in the scanner data, multiplied by the level of consumption reflected in the post-tax environment for each county. This inherently assumes that the tax

itself produces a deadweight loss of market inefficiency that amounts to a societal cost [20,21]. Costs and QALYs were integrated over the life-course at a 3% discount rate. The process was repeated 10,000 times across the simulated Oakland population, with 95% credible intervals calculated around each outcome variable computed from the outcomes upon repeated Monte Carlo sampling from the distribution of each input variable.

Microsimulation Estimates

The simulated Oakland population, based on the ACS (2019), had a median age of 36.9 years old, with 66% of the population aged 18 to 64; 52% male; and 29% White, 24% Black, 14% Asian, and 27% Hispanic (including all races under Hispanic ethnicity; **S6 Table**). The mean age-adjusted lifetime was 83.1 years for Oakland.

The simulated population, based on NHANES populations sampled and weighted to reflect the American Community Survey demographics of Alameda County, had a 17.4% prevalence of obesity, 5.2% prevalence of coronary heart disease, 3.0% prevalence of cerebrovascular accident, 7.9% prevalence of diabetes, 11.3% prevalence of chronic kidney disease, and 82.1% prevalence of dental disease (**S6 Table**); the first four statistics were possible to validate against the California Health Interview Survey (CHIS), and were found to be within 0.1% absolute percentage points of estimates from CHIS for Alameda County (2019) [22].

The simulated Oakland population, without the SSB tax, would be expected to accrue a discounted 63,382 QALYs over 10 years (95% CI 63,289 to 63,482; or 0.63 QALYs per person per year of life) and 270,385 over a lifetime (95% CI 269,535 to 271,340; or 0.60 QALYs per person per year of life) per 10,000 population (overall lower quality of life than San Francisco's population due to a higher disease burden). The population would, in parallel, be expected to accrue $21,799,779 per 10,000 people over 10 years for the studied health outcomes (95% CI $21,653,301 to $21,935,634; or $218 per person per year), and $240,390,320 per 10,000 over a lifetime (95% CI $238,519,358 to $242,331,645; or $529 per person per year; a lower cost than

San Francisco's population due to lower rate of commercial insurance and more public insurance coverage).

The simulated Oakland population, in the context of the SSB tax, was estimated to reduce SSB consumption by 26.8%, or 1.33 ounces per person per day. The reduced intake was estimated to accrue 94.0 QALYs over 10 years (95% CI 5.2 to 235.8; 0.0009 QALYs per person per year) and 967.6 QALYs over a lifetime (95% CI 70.4 to 2446.0; 0.0022 QALYs per person per year) per 10,000 population, with a roughly even distribution of gains across the disease outcomes studied (Table 3, Figure 2). From a healthcare perspective, cost savings were estimated at $106,108 over 10 years (95% CI $10,739 to $251,543; $1.06 per person per year) and $1,269,770 over a lifetime (95% CI $96,159 to $3,163,781; $2.85 per person per year) per 10,000 people, with dental disease being among the top short-term drivers of cost savings and coronary heart disease and chronic kidney disease among the top longer-term drivers. From a societal perspective, incorporating the cost of the SSB tax itself, the net tax impact per 10,000 people was an estimated cost savings of $102,154 over 10 years (95% CI $6,785 to $247,589; $1.02 per person per year) and $1,251,817 over a lifetime (95% CI $78,206 to $3,145,828; $2.81 per person per year).

Point of Cost Neutrality

The estimates regarding cost savings were robust to a variety of substitution scenarios. For the calculated cost savings to be invalidated ("neutralized"), the measured association of the SSB tax with purchases would need to overestimate the actual relationship (e.g., as a result of hidden cross-border purchasing, caloric substitution and familial/household consumption patterns) to a large degree. The long-term cost savings would be fully negated if the estimated reduction in SSB purchases resulted in a reduction in actual intake of only 0.3% in Oakland. In other words, this would require overestimating the change in actual SSB intake by 98-99% in either city.

SUPPLEMENTAL REFERENCES

1. Wing C, Simon K, Bello-Gomez RA. Designing Difference in Difference Studies: Best Practices for Public Health Policy Research. Annual Review of Public Health. 2018;39:453-69. doi: 10.1146/annurev-publhealth-040617-013507.
2. Wooldridge JM. Econometric analysis of cross section and panel data: MIT Press; 2010.
3. Anderson ML. Multiple Inference and Gender Differences in the Effects of Early Intervention: A Reevaluation of the Abecedarian, Perry Preschool, and Early Training Projects. J Am Stat Assoc. 2008;103(484):1481-95. doi: 10.1198/016214508000000841.
4. Abadie A. Using Synthetic Controls: Feasibility, Data Requirements, and Methodological Aspects. J Econ Lit. 2021;59(2):391-425. doi: 10.1257/jel.20191450.
5. Abadie A, Diamond A, Hainmueller J. Synthetic control methods for comparative case studies: Estimating the effect of California's tobacco control program. J Am Stat Assoc. 2010;105(490):493-505.
6. Altman DG, Bland JM. How to obtain the confidence interval from a P value. BMJ. 2011;343:d2090. doi: 10.1136/bmj.d2090.
7. Cavallo E, Galiani S, Noy I, Pantano J. Catastrophic Natural Disasters and Economic Growth. The Review of Economics and Statistics. 2013;95(5):1549-61. doi: 10.1162/REST_a_00413.
8. Basu S, Jacobs LM, Epel E, Schillinger D, Schmidt L. Cost-effectiveness of a workplace ban on sugar-sweetened beverage sales: A microsimulation model. Health Aff (Millwood). 2020;39(7):1140-8. doi: 10.1377/hlthaff.2019.01483. PubMed PMID: 32634357.
9. Visscher TL, Snijder MB, Seidell JC. Epidemiology: Definition and classification of obesity. In: Kopelman PG, Caterson ID, Dietz WH, editors. Clinical obesity in adults and children. Hoboken (NJ): Wiley; 2009. p. 3-14.
10. Sánchez-Romero LM, Penko J, Coxson PG, Fernández A, Mason A, Moran AE, et al. Projected Impact of Mexico's Sugar-Sweetened Beverage Tax Policy on Diabetes and Cardiovascular Disease: A Modeling Study. PLoS Med. 2016;13(11):e1002158. doi: 10.1371/journal.pmed.1002158. PubMed Central PMCID: PMCPMC5089730
11. Sacco RL, Kasner SE, Broderick JP, Caplan LR, Connors J, Culebras A, et al. An updated definition of stroke for the 21st century: A statement for healthcare professionals from the American Heart Association/American Stroke Association. Stroke. 2013;44(7):2064-89.
12. American diabetes Association. 2. Classification and Diagnosis of Diabetes: Standards of Medical Care in Diabetes—2021. Diabetes Care. 2021;44(Supplement 1):S15-S33. doi: 10.2337/dc21-S002.
13. Levey AS, Coresh J, Bolton K, Culleton B, Harvey KS, Ikizler TA, et al. K/DOQI clinical practice guidelines for chronic kidney disease: Evaluation, classification, and stratification. Am J Kidney Dis. 2002;39(2, Suppl 1):S1-S266.
14. Matsushita K, Mahmoodi BK, Woodward M, Emberson JR, Jafar TH, Jee SH, et al. Comparison of Risk Prediction Using the CKD-EPI Equation and the MDRD Study Equation for Estimated Glomerular Filtration Rate. JAMA. 2012;307(18):1941-51. doi: 10.1001/jama.2012.3954.

15. Eke PI, Dye BA, Wei L, Slade GD, Thornton-Evans GO, Borgnakke WS, et al. Update on Prevalence of Periodontitis in Adults in the United States: NHANES 2009 to 2012. J Periodontol. 2015;86(5):611-22. doi: https://doi.org/10.1902/jop.2015.140520.
16. Institute for Health Metrics and Evaluation. Global Burden of Disease Results Tool Seattle, Washington: University of Washington; 2021 [Accessed on January 21, 2021]. Available from: http://ghdx.healthdata.org/gbd-results-tool.
17. Husereau D, Drummond M, Petrou S, Carswell C, Moher D, Greenberg D, et al. Consolidated Health Economic Evaluation Reporting Standards (CHEERS)—Explanation and Elaboration: A Report of the ISPOR Health Economic Evaluation Publication Guidelines Good Reporting Practices Task Force. Value Health. 2013;16(2):231-50. doi: https://doi.org/10.1016/j.jval.2013.02.002.
18. van Buuren S, Groothuis-Oudshoorn K. mice: Multivariate Imputation by Chained Equations in R. Journal of Statistical Software. 2011;45(3):1 - 67. doi: 10.18637/jss.v045.i03.
19. National Cancer Institute Division of Cancer Control & Population Sciences. Usual Dietary Intakes: SAS Macros for Analysis of a Single Dietary Component 2021 [cited 2021 January 21, 2021]. Available from: https://epi.grants.cancer.gov/diet/usualintakes/macros_single.html.
20. Hausman JA. Exact Consumer's Surplus and Deadweight Loss. Am Econ Rev. 1981;71(4):662-76.
21. Diewert WE. The Measurement of Deadweight Loss Revisited. Econometrica. 1981;49(5):1225-44. doi: 10.2307/1912752.
22. California Health Interview Survey. AskCHIS Los Angeles, CA: University of California Los Angeles Center for Helath Policy Research; 2021 [cited 2021 January 21, 2021]. Available from: http://ask.chis.ucla.edu/.
