# Supplementary material for: Evaluation of the sugar-sweetened beverage tax in Oakland, United States, 2015–2019: A quasi-experimental and cost-effectiveness study
Source: PLoS Med. 2023 Apr 18;20(4):e1004212. doi: 10.1371/journal.pmed.1004212 (PMC10112812; doi:10.1371/journal.pmed.1004212)
Supplement: S1 Prospective Plan — (PDF) [file pmed.1004212.s003.pdf]

S1 Prospective Plan

This document presents our prospective analytic plan as described in the NIH grant proposal (Award Number R01 DK116852) that funded this work.

Data Sources

We will use retail scanner data that can be merged with socio-demographic information at the zip code level to perform the primary analysis described below. The data include quad-weekly prices and sales for sugar-sweetened beverages (SSBs) and other beverages from 2015-2019 by store, product, and location with store identifiers, purchased from Information Resources, Inc (IRi).

Analytic Plan

Given changing trends in SSB consumption at the national level, we will include appropriate comparator groups to account for secular trends. Based on our preliminary analyses, we will use Los Angeles (LA) and Richmond as comparison cities using IRi scanner data. Demographics of the two cities are quite similar to Oakland, as is SSB consumption in the period prior to the SSB tax. Final selection of the comparator group will be made after 2016 scanner data become available for incorporation in our preliminary analysis.

Two statistical analyses will be conducted.

First, we will perform standard difference-in-differences (DD) analysis, which compares the outcome metrics in tax-affected (Oakland) to -unaffected (Richmond/LA) populations, before and after the tax. The DD model specification is: $Y_{ipt} = \beta D_{it} + \alpha_i + \rho_p + \theta_t + \varepsilon_{ipt}$, where outcome metric Y_{ipt} for city *i* at time *t*; β is the difference-in-differences treatment effect of interest; the treatment indicator D_{it} equals 1 if city *i* has been exposed to the tax at time *t* and 0 otherwise; α_i, ρ_p, and θ_t are city, product (UPC), and time fixed effects to net out time-invariant unobserved differences between the cities, products, and secular trends in purchases

common throughout the region, and ε_{ipt} is the error term. Standard errors will be clustered by zip code to account for correlated outcomes within zip code.

While overall SSB purchases is our primary outcome of interest, we will create separate models for each beverage category to explore changes across taxed and untaxed beverage categories (capturing substitution effects). We will also explore changes in “junk food” such as cookies and donuts as a further compensatory response to the tax. We will estimate models by store channel to identify the types of stores that experience the larger change in sales. Tax-affected individuals could purchase in neighboring areas to avoid the tax. Conversely, persons in adjacent areas may reduce their intake as a result of exposure to the media campaigns and changes in social norms related to the tax. In a secondary analysis using the scatter data, we will conduct a DD analysis to compare changes in neighboring jurisdictions to Oakland against neighboring jurisdictions to our comparator cities (Richmond and LA). The DD analysis will help determine whether people have been shifting purchases of taxed beverages to neighboring cities, as nearby stores in untaxed areas would be shown to have increased sales, net of secular trends.

In a second method, we will perform a synthetic control analysis using the IRI scanner data. To select our comparison group in an algorithmic way, we will construct a synthetic control for the tax city as a weighted average of all possible stores in the comparator area, based on the combination of stores that provide the best fit for the pre-tax trend. Following established procedures adopted in seminal work, we will assign weights to the pool of potential control stores to generate a synthetic control. This entails minimizing the mean squared prediction error to find the best match to pre-tax trends in each tax city. We will then conduct a placebo or falsification test for stores that were not exposed to an SSB tax, similar to a Fisher exact test. We will observe the proportion of control stores with an estimated change in SSB sales as extreme or more extreme than in Oakland. This will indicate how statistically significant the estimated effects in Oakland are compared with control stores.

Statistical power. With an estimated sample size of 42,120 store-month observations (2015-2019), we have ≥80% power to detect differences in purchases of SSBs per store of <0.5%, with two-sided alpha of 0.05. This effect is extremely low and far smaller than observed for SSB taxes in Berkeley (21% decline) and Mexico (12% decline), and will critically help to assess sustained effects over time.
