# Supplementary material for: Evaluation of the sugar-sweetened beverage tax in Oakland, United States, 2015–2019: A quasi-experimental and cost-effectiveness study
Source: PLoS Med. 2023 Apr 18;20(4):e1004212. doi: 10.1371/journal.pmed.1004212 (PMC10112812; doi:10.1371/journal.pmed.1004212)
Supplement: S1 Table — (PDF) [file pmed.1004212.s004.pdf]

S1 Table. Socio-demographic characteristics by city

	Oakland		Richmond
	All areas	Low-income areas [a]	All areas
Population	381,459	187,606	81,602
Aged 18 to 64 (%)	67.0	65.9	64.1
White (%)	35.6	22.3	33.3
Black (%)	26.5	28.3	25.6
Asian (%)	17.3	20.6	9.4
Other/multi race (%)	21.4	29.8	32.3
Hispanic (%)	25.4	38.4	44.4
Median household income ($)	65,503	38,648	55,559
Household income <$10K (%)	6.9	10.0	7.0
Household income <$25K (%)	23.9	34.5	24.5
Household income <$50K (%)	43.5	58.7	48.5

[a] Zip codes where household income is below the city's median.

Note: Data are drawn from the 2010 US decennial census (population, age, race/ethnicity) and the 2016 American Community Survey (income).
