# Supplementary material for: Evaluation of the sugar-sweetened beverage tax in Oakland, United States, 2015–2019: A quasi-experimental and cost-effectiveness study
Source: PLoS Med. 2023 Apr 18;20(4):e1004212. doi: 10.1371/journal.pmed.1004212 (PMC10112812; doi:10.1371/journal.pmed.1004212)
Supplement: S2 Table — (PDF) [file pmed.1004212.s005.pdf]

S2 Table. Volume sales per store before and after the Oakland tax in a panel of stores and products in Oakland and Richmond (a comparator)

	Intervention (with tax)		Comparator (no tax)	
Outcome: volume sales, in 100s oz[a]	Pre[b]	Post[c]	Pre[b]	Post[c]
All beverages				
SSBs	1063.06	1001.93	865.08	935.11
Untaxed beverages	1927.66	1813.29	1085.71	1028.87
SSBs by beverage category				
Soda	206.09	199.07	199.58	193.65
Fruit drinks	200.25	199.37	134.54	141.00
Sports drinks	366.26	333.12	329.20	397.33
Energy drinks	8.70	6.59	9.25	8.89
Coffee	45.45	51.34	48.34	51.14
Tea	184.18	157.76	112.14	105.00
Flavored water	1.90	1.97	4.41	3.17
SSBs by store type				
Convenience stores	398.14	398.44	421.78	443.29
Pharmacies	357.45	344.77	344.47	340.00
Supermarkets	3001.20	2778.84		
SSBs by beverage size[d]				
Individual	618.82	597.73	544.19	642.94
Family	443.73	404.44	318.98	297.51
SSBs by income[e]				
Stores in lower income area	920.41	988.90	1399.09	1503.41
Stores in higher income area	1243.57	1009.36	352.65	366.81
SSBs in border areas[f]	1744.49	1870.12	2146.79	2222.18
Sweet snacks[g]	0.95	0.93	1.05	0.94

[a] Mean monthly volume, in 100s of fl oz, for all beverage products sold in a store. For sweet snacks, volume is measured in 100s of ounces. The sample is restricted to a panel of stores and products with data available throughout the study period.

[b] Unadjusted volume. The before-tax period for the Oakland tax is January 1, 2015 through June 30, 2017.

[c] Unadjusted volume. The after-tax period for the Oakland tax is July 1, 2017 through December 31, 2019.

[d] Beverages are individual-sized if 36 fl oz or less and family-sized if more than 36 fl oz.

[e] Income was based on zip code-level data from 2016 5-year American Community Survey estimates. Zip codes with a below-median proportion of residents living under $35,000 were considered a lower income area, and zip codes with an above-median proportion of residents living under $35,000 were considered a higher income area.

[f] This compares border zip codes around Oakland to the border zip codes around Richmond.

[g] This includes all products within the doughnuts and cookies categories.
