# Supplementary material for: Evaluation of the sugar-sweetened beverage tax in Oakland, United States, 2015–2019: A quasi-experimental and cost-effectiveness study
Source: PLoS Med. 2023 Apr 18;20(4):e1004212. doi: 10.1371/journal.pmed.1004212 (PMC10112812; doi:10.1371/journal.pmed.1004212)
Supplement: S3 Table — (PDF) [file pmed.1004212.s006.pdf]

S3 Table. Difference-in-differences estimates of change in volume sales of untaxed beverages in Oakland compared with Richmond

Outcome: volume sales, in 100s oz[a]	Intervention (with tax)		Comparator (no tax)		Adjusted % Change[d]	Adjusted Difference-in-Differences Estimate	
	Pre[b]	Post[c]	Pre[b]	Post[c]		Coef. (95% CI)	*p*-value
All untaxed beverages	1927.66	1813.29	1085.71	1028.87	-4.91	-154.99 (-584.86 to 274.88)	0.48
By beverage category							
Soda	69.27	70.34	68.74	69.10	16.00	1973.08 (-4837.14 to 8783.29)	0.57
Fruit drinks	937.11	808.89	414.13	342.44	-0.08	-105.96 (-13505.68 to 13293.76)	0.99
Sports drinks	73.99	56.67	55.36	59.31	-37.55	-5530.73 (-8422.45 to -2639.01)	<0.001
Energy drinks	10.46	9.00	12.71	10.91	8.16	85.99 (-382.96 to 554.93)	0.72
Coffee	6.39	16.66	7.07	15.23	26.08	212.51 (-705.33 to 1130.35)	0.65
Tea	215.14	186.58	128.97	88.69	-6.02	-1626.47 (-5518.65 to 2265.71)	0.41
Flavored water	8.55	20.02	8.11	24.12	-45.13	-643.29 (-3055.38 to 1768.80)	0.60
Club soda/tonic water	2.16	2.39	0.46	0.54	-6.05	-14.40 (-52.76 to 23.96)	0.46
Bottled water	30.73	44.12	27.18	30.18	6.29	332.46 (-282.01 to 946.93)	0.29
100% fruit juice	268.19	311.89	198.41	214.38	-32.01	-13264.19 (-27576.93 to 1048.54)	0.07
Diet soda	3.48	3.37	2.51	2.49	-8.25	-28.03 (-105.52 to 49.46)	0.48
Milk	164.43	158.52	126.63	116.09	-4.32	-763.29 (-1843.91 to 317.34)	0.17
Milk alternatives	16.95	20.76	9.12	10.45	2.75	51.10 (-217.21 to 319.41)	0.71
By store type							
Convenience stores	165.98	165.27	171.64	156.51	3.28	5.75 (-36.93 to 48.43)	0.79
Pharmacies	473.77	389.78	431.88	353.42	-5.51	-29.06 (-153.18 to 95.07)	0.65
Supermarkets	6337.11	6046.07			10.04	1013.41 (240.57 to 1786.25)	0.01
By beverage size[e]							
Individual	439.07	428.65	270.28	295.28	-15.56	-83.94 (-146.75 to -21.13)	0.009
Family	1488.59	1384.64	815.43	733.58	-3.03	-75.70 (-409.32 to 257.91)	0.66
By area income[f]							
Stores in lower income area	1617.31	1786.16	1865.26	1773.26	-4.87	-110.83 (-229.54 to 7.89)	0.07
Stores in higher income area	2311.03	1846.80	337.66	284.47	-4.26	-173.55 (-657.68 to 310.57)	0.48
In border area[g]	2848.39	2760.71	4063.29	3762.53	3.31	128.71 (-293.43 to 550.85)	0.55

[a] Mean monthly beverage volume, in 100s of fl oz, for all beverage products sold in a store. The sample is restricted to a panel of stores and products with data available throughout the study period.

[b] Unadjusted volume. The before-tax period for the Oakland tax is January 1, 2015 through June 30, 2017.

[c] Unadjusted volume. The after-tax period for the Oakland tax is July 1, 2017 through December 31, 2019.

[d] Percent change and difference-in-differences estimates are based on regression analyses. The percent change was calculated by dividing the difference-in-difference estimate by the average pre-tax volume in Oakland. The numerator represents the change in volume sales in the post-tax period compared with the pre-tax period controlling for secular trends using Richmond as a comparator.

[e] Beverages are individual-sized if 36 fl oz or less and family-sized if more than 36 fl oz.

[f] Income was based on zip code-level data from 2016 5-year American Community Survey estimates. Zip codes with a below-median proportion of residents living under $35,000 were considered a lower income area, and zip codes with an above-median proportion of residents living under $35,000 were considered a higher income area.

[g] This compares border zip codes around Oakland to the border zip codes around Richmond.
