# Supplementary material for: Evaluation of the sugar-sweetened beverage tax in Oakland, United States, 2015–2019: A quasi-experimental and cost-effectiveness study
Source: PLoS Med. 2023 Apr 18;20(4):e1004212. doi: 10.1371/journal.pmed.1004212 (PMC10112812; doi:10.1371/journal.pmed.1004212)
Supplement: S4 Table — (PDF) [file pmed.1004212.s007.pdf]

S4 Table. Test of parallel pre-trends in volume sales between Oakland and Richmond in event study difference-in-differences model

	Fraction of pre-tax DiD coefficients significant at p<0.05
All beverages	
SSBs	0/8
Untaxed beverages	0/8
SSBs by category	
Soda	1/8
Fruit drinks	1/8
Sports drinks	0/8
Energy drinks	0/8
Coffee	5/8
Tea	2/8
Flavored water	1/8
SSBs by store type	
Mass merchandise stores	
Convenience stores	1/8
Pharmacies	1/8
Supermarkets	4/8
SSBs by beverage size	
Individual	0/8
Family	0/8
SSBs by income	
Stores in lower income area	2/8
Stores in higher income area	3/8
SSBs in border areas	1/8
Sweet snacks	0/8

Note: This table shows the fraction of difference-in-differences (DiD) coefficients on each before-tax quarter that are statistically significant at the 0.05 level, according to event study difference-in-differences estimates of the change in volume sales.
