# Supplementary material for: Evaluation of the sugar-sweetened beverage tax in Oakland, United States, 2015–2019: A quasi-experimental and cost-effectiveness study
Source: PLoS Med. 2023 Apr 18;20(4):e1004212. doi: 10.1371/journal.pmed.1004212 (PMC10112812; doi:10.1371/journal.pmed.1004212)
Supplement: S5 Table — (PDF) [file pmed.1004212.s008.pdf]

**S5 Table.** Synthetic control estimates of the association between the Oakland tax and SSB volume sales in the Oakland border area

| Model specification | Coef. (95% CI) |
| --- | --- |
| **Base model:** | |
| 1. LA-only donor pool | 0.041 (-0.062 to 0.145) |
| | |
| **Sensitivity checks:** | |
| 2. LA and Richmond donor pool | 0.091 (-0.004 to 0.186) |
| 3. Adjust for retail price per oz | 0.039 (-0.015 to 0.094) |
| 4. Cross-validation | 0.020 (-0.019 to 0.059) |
| 5. Panel of UPCs | 0.090 (-0.036 to 0.215) |

Note: Each row represents a different set of models. Coefficient is calculated as the mean estimate of the association between the taxes and volume sales during the post-tax period.
