# Supplementary material for: Evaluation of the sugar-sweetened beverage tax in Oakland, United States, 2015–2019: A quasi-experimental and cost-effectiveness study
Source: PLoS Med. 2023 Apr 18;20(4):e1004212. doi: 10.1371/journal.pmed.1004212 (PMC10112812; doi:10.1371/journal.pmed.1004212)
Supplement: S6 Table — (PDF) [file pmed.1004212.s009.pdf]

S1 Table. Summary statistics on model input data

Parameter(s)	Value(s) [Standard deviation]	Data source
Age, years	Median 36.9 (66% aged 18-64 years old)	ACS for Oakland
Sex, % male	52%	ACS for Oakland
Race/ethnicity	29% White, 24% Black, 14% Asian, 27% Hispanic	ACS for Oakland
Baseline disease prevalence	17.4% [0.8%] obesity,<br>5.2% [0.2%] coronary heart disease,<br>3.0% [0.1%] history of cerebrovascular accident,<br>7.9% [0.3%] diabetes mellitus,<br>11.3% [0.5%] chronic kidney disease,<br>82.1% [3.6%] dental disease	NHANES weighted to ACS demographics for Oakland (see age, sex, and race-specific estimates in Appendix)
Disease incidence, per 10,000 per year*	153.6 [6.8] coronary heart disease,<br>80.9 [3.6] cerebrovascular accident,<br>338.3 [15.0] diabetes mellitus,<br>333.8 [14.8] chronic kidney disease,<br>57790.0 [2568.4] dental disease	GBD (see age and sex-specific estimates in Appendix)
Mortality, per 10,000 per year	56.3 [2.5] obesity,<br>78.4 [3.5] coronary heart disease,<br>26.2 [1.2] cerebrovascular accident,<br>11.6 [0.5] diabetes mellitus,<br>15.1 [0.7] chronic kidney disease,<br><1 [0.1] dental disease,<br>442.3 [19.7] total all-cause	GBD (see age and sex-specific estimates in Appendix)
Healthcare costs per outcome per year, US$2021[†]	Obesity: $1861 (Medicare) [652], $1103 (Medicaid) [723], $1231 (commercial) [204], $1547 (uninsured) [246];<br>Coronary heart disease: $4959 [531] (Medicare), $4690 [531] (Medicaid), $4677 [650] (commercial), $2650 [441] (uninsured);<br>Cerebrovascular accident: $8494 [1021]] (Medicare), $8443 [1021] (Medicaid), $9863 [5081] (commercial), $222 [2419] (uninsured);<br>Diabetes mellitus: $3528 [408] (Medicare), $4524 [408] (Medicaid), $3489 [300] (commercial), $1496 [249] (uninsured);<br>Chronic kidney disease: $5965 [504] (Medicare), $14864 [504] (Medicaid), $5453 [1,845] (commercial), $3951 [1,028] (uninsured)	MEPS (see standard errors around estimates in Appendix)

	Dental disease: $132 [210] (Medicare), $340 [210] (Medicaid), $320 [124] (commercial), $58 [101] (uninsured)	
Baseline SSB consumption, ounces per person per day	4.95 [26.83]	NHANES weighted to ACS demographics for Oakland (see age, sex, and race-specific estimates in Appendix)
SSB consumption reduction, ounces per person per day, attributable to tax	1.33 [0.60]	Current study
Change in outcome risk, per each 1 ounce/person/day decline in SSB consumption from ban**	0.53 weight (kg) change [0.08],<br>0.98 RR coronary heart disease [0.02],<br>0.99 RR cerebrovascular accident [0.01],<br>0.97 RR diabetes mellitus [0.03],<br>0.98 RR chronic kidney disease [0.02],<br>0.98 RR dental disease [0.02],<br>0.99 RR all-cause mortality [0.01]	Rebholz et al. (2019),[1] Imamura et al. (2015),[2] Bernabé et al. (2014),[3] Malik et al. (2019),[4] Hall et al. (2011),[5] Wang et al. (2012)[6]
Disutility used for quality-adjusted life-year calculations	0.85 obesity [0.13],<br>0.89 coronary heart disease [0.04],<br>0.69 cerebrovascular accident [0.03],<br>0.96 diabetes mellitus [0.04],<br>0.99 chronic kidney disease [0.01],<br>0.99 dental disease [0.01]	Salomon et al. (2015),[7] Craig and Tseng (2002)[8]

Note: ACS: American Community Survey (2019); NHANES: National Health and Nutrition Examination Survey (2015-2018); GBD: Global Burden of Disease Study (2019); MEPS: Medical Expenditure Panel Survey (2018). RR: relative risk. SSB: sugar-sweetened beverage. To estimate uncertainty in the outcomes, repeated Monte Carlo sampling was performed from the uncertainty distributions (Gaussian distributions built from the 95% confidence intervals around the estimates).

* for obesity, linear secular trends in weight by age were used from NHANES, as GBD data were limited to annual percent changes in obesity rather than incidence.

† for obesity, costs were obtained from Finkelstein et al. (2009),[9] as these costs were not directly available from MEPS.

** weight change is change in kilograms over 3 years from the reduction in sugar-sweetened beverage consumption. Other estimates are relative risk in incidence/mortality.

S6 Table References

1. Rebholz CM, Young BA, Katz R, Tucker KL, Carithers TC, Norwood AF, et al. Patterns of Beverages Consumed and Risk of Incident Kidney Disease. Clin J Am Soc Nephrol. 2019;14(1):49-56. doi: 10.2215/cjn.06380518.
2. Imamura F, O'Connor L, Ye Z, Mursu J, Hayashino Y, Bhupathiraju SN, et al. Consumption of sugar sweetened beverages, artificially sweetened beverages, and fruit juice and incidence of type 2 diabetes: systematic review, meta-analysis, and estimation of population attributable fraction. BMJ. 2015;351:h3576. Epub 2015/07/23. doi: 10.1136/bmj.h3576. PubMed PMID: 26199070; PubMed Central PMCID: PMCPMC4510779.
3. Bernabé E, Vehkalahti MM, Sheiham A, Aromaa A, Suominen AL. Sugar-sweetened beverages and dental caries in adults: A 4-year prospective study. J Dent. 2014;42(8):952-8. doi: https://doi.org/10.1016/j.jdent.2014.04.011.
4. Malik VS, Li Y, Pan A, Koning LD, Schernhammer E, Willett WC, et al. Long-Term Consumption of Sugar-Sweetened and Artificially Sweetened Beverages and Risk of Mortality in US Adults. Circulation. 2019;139(18):2113-25. doi: doi:10.1161/CIRCULATIONAHA.118.037401.
5. Hall KD, Sacks G, Chandramohan D, Chow CC, Wang YC, Gortmaker SL, et al. Quantification of the effect of energy imbalance on bodyweight. Lancet. 2011;378(9793):826-37. doi: https://doi.org/10.1016/S0140-6736(11)60812-X.
6. Wang YC, Coxson P, Shen Y-M, Goldman L, Bibbins-Domingo K. A penny-per-ounce tax on sugar-sweetened beverages would cut health and cost burdens of diabetes. Health Aff (Millwood). 2012;31(1):199-207. PubMed PMID: 22232111.
7. Salomon JA, Haagsma JA, Davis A, de Noordhout CM, Polinder S, Havelaar AH, et al. Disability weights for the Global Burden of Disease 2013 study. Lancet Glob Health. 2015;3(11):e712--e23. doi: 10.1016/S2214-109X(15)00069-8.
8. Craig BM, Tseng DS. Cost-effectiveness of gastric bypass for severe obesity. Am J Med. 2002;113(6):491-8. doi: https://doi.org/10.1016/S0002-9343(02)01266-4.
9. Finkelstein EA, Trogdon JG, Cohen JW, Dietz W. Annual Medical Spending Attributable To Obesity: Payer-And Service-Specific Estimates. Health Aff (Millwood). 2009;28(Supplement 1):w822-w31. doi: 10.1377/hlthaff.28.5.w822.
