# Supplementary material for: Evaluation of the sugar-sweetened beverage tax in Oakland, United States, 2015–2019: A quasi-experimental and cost-effectiveness study
Source: PLoS Med. 2023 Apr 18;20(4):e1004212. doi: 10.1371/journal.pmed.1004212 (PMC10112812; doi:10.1371/journal.pmed.1004212)
Supplement: S7 Table — (PDF) [file pmed.1004212.s010.pdf]

S7 Table. Population-weighted estimates from the National Health and Nutrition Examination Study (2015-2018), sampled to represent the population of Alameda county

Age, yrs	Sex	Race/ethnicity	n	Obese (%)	Coronary heart disease (%)	Cerebrovascular disease (%)	Diabetes (%)	Chronic kidney disease (%)	Dental disease (%)	Non-zero SSB consumption(%)	SSB consumption kcal/person/day, among those with non-zero consumption [IQR]
<8	Male	non-Hispanic White	5317467.2	12531.2 (0.3)	0.0 (NaN)	0.0 (NaN)	21129.9 (0.4)	0.0 (NaN)	0.0 (NaN)	775354.9 (14.6)	49.60 [25.00, 80.38]
8-<18	Male	non-Hispanic White	7725596.8	431778.6 (6.0)	0.0 (NaN)	0.0 (NaN)	109560.2 (1.4)	27427.4 (0.7)	0.0 (NaN)	3573385.2 (46.3)	110.00 [74.50, 184.06]
18-<40	Male	non-Hispanic White	17073578.1	5641086.0 (36.1)	200950.7 (1.3)	40614.8 (0.3)	490652.2 (2.9)	2209637.5 (14.8)	890159.3 (97.5)	6258184.2 (36.7)	152.00 [78.00, 280.00]
40-<65	Male	non-Hispanic White	21318587.5	9282521.5 (45.6)	1877379.1 (8.8)	491110.4 (2.3)	3591028.4 (16.8)	9219757.9 (46.3)	1122902.1 (97.3)	6835026.9 (32.1)	156.00 [78.00, 290.50]
≥65	Male	non-Hispanic White	10865730.2	4055727.4 (40.4)	2746250.8 (25.3)	877813.7 (8.1)	3465619.8 (31.9)	8677406.1 (88.6)	420788.5 (95.3)	2332615.2 (21.5)	89.68 [61.00, 155.78]
<8	Female	non-Hispanic White	5037883.6	0.0 (0.0)	0.0 (NaN)	0.0 (NaN)	0.0 (0.0)	0.0 (NaN)	0.0 (0.0)	697434.1 (13.8)	45.44 [22.42, 83.86]
8-<18	Female	non-Hispanic White	7267893.6	442286.9 (6.5)	0.0 (NaN)	0.0 (NaN)	7774.6 (0.1)	15977.6 (0.4)	29976.5 (100.0)	2418719.4 (33.3)	78.00 [50.39, 149.00]
18-<40	Female	non-Hispanic White	16752266.5	5640007.3 (34.8)	196131.0 (1.3)	41827.7 (0.3)	582352.6 (3.5)	2297686.9 (15.0)	527506.5 (72.8)	5041888.0 (30.1)	149.21 [78.00, 228.58]
40-<65	Female	non-Hispanic White	22318026.0	9089308.0 (42.3)	896475.6 (4.0)	516765.9 (2.3)	2743038.9 (12.3)	9408859.7 (45.2)	840547.3 (98.8)	5836228.4 (26.2)	98.69 [56.36, 209.40]
≥65	Female	non-Hispanic White	13600567.3	5158210.1 (41.1)	2068039.1 (15.2)	1150692.6 (8.5)	3026180.1 (22.3)	10763541.0 (88.5)	171539.4 (100.0)	2373525.4 (17.5)	78.23 [52.00, 137.33]
<8	Male	non-Hispanic Black	1382099.4	0.0 (0.0)	0.0 (NaN)	0.0 (NaN)	0.0 (0.0)	0.0 (NaN)	0.0 (NaN)	190158.8 (13.8)	36.72 [23.28, 68.50]
8-<18	Male	non-Hispanic Black	1973268.1	234568.5 (12.6)	0.0 (NaN)	0.0 (NaN)	7449.4 (0.4)	0.0 (0.0)	4992.7 (100.0)	749788.1 (38.0)	79.72 [56.00, 149.99]
18-<40	Male	non-Hispanic Black	3727607.2	1029153.9 (29.6)	52720.7 (1.5)	24244.8 (0.7)	167951.8 (4.5)	299503.6 (9.6)	175478.3 (85.0)	1467420.4 (39.4)	130.87 [78.00, 221.00]
40-<65	Male	non-Hispanic Black	3536161.3	1647409.4 (48.7)	250094.6 (7.1)	160557.4 (4.5)	835502.9 (23.6)	1472901.0 (47.1)	397970.8 (90.0)	1384921.7 (39.2)	136.77 [78.00, 234.00]
≥65	Male	non-Hispanic	1091055.8	327496.1 (32.8)	259990.5 (23.8)	132840.1 (12.2)	445975.4 (40.9)	693165.5 (75.3)	91350.7 (100.0)	346707.6 (31.8)	97.49 [69.35,

		Black									163.32]
<8	Female	non-Hispanic Black	1312795.2	3948.3 (0.4)	0.0 (NaN)	0.0 (NaN)	2752.1 (0.2)	0.0 (NaN)	0.0 (NaN)	168935.5 (12.9)	51.08 [22.62, 83.14]
8-<18	Female	non-Hispanic Black	2010151.7	353222.1 (18.6)	0.0 (NaN)	0.0 (NaN)	43293.5 (2.2)	0.0 (0.0)	3641.9 (35.8)	701497.0 (34.9)	78.19 [51.00, 127.30]
18-<40	Female	non-Hispanic Black	4240327.2	1993164.3 (49.3)	27028.8 (0.7)	55063.7 (1.4)	228182.4 (5.4)	165957.2 (4.6)	131163.3 (94.4)	1832588.9 (43.2)	109.35 [68.61, 192.18]
40-<65	Female	non-Hispanic Black	4419320.1	2582020.5 (61.2)	345389.7 (7.8)	262910.8 (5.9)	988180.2 (22.4)	1226978.2 (31.5)	204505.9 (100.0)	1806840.6 (40.9)	102.27 [64.33, 179.53]
≥65	Female	non-Hispanic Black	1570052.5	783121.6 (53.9)	277919.1 (17.7)	153594.9 (9.8)	561382.8 (35.8)	1026139.3 (77.5)	71813.5 (100.0)	425177.7 (27.1)	93.87 [62.00, 155.51]
<8	Male	Hispanic	2759359.0	0.0 (0.0)	0.0 (NaN)	0.0 (NaN)	5520.1 (0.2)	0.0 (NaN)	0.0 (NaN)	470095.8 (17.0)	38.56 [21.78, 66.95]
8-<18	Male	Hispanic	3422050.8	415276.8 (12.5)	0.0 (NaN)	0.0 (NaN)	6034.2 (0.2)	0.0 (0.0)	19500.5 (60.1)	1497514.4 (43.8)	78.00 [51.00, 121.93]
18-<40	Male	Hispanic	6735657.2	2696694.5 (44.1)	20555.2 (0.3)	23743.6 (0.4)	275694.5 (4.1)	460698.1 (7.9)	263577.9 (92.0)	3299671.2 (49.0)	152.33 [78.00, 239.12]
40-<65	Male	Hispanic	4998276.8	2181909.0 (45.4)	282724.6 (5.7)	116538.2 (2.3)	1153592.7 (23.1)	1430954.1 (30.8)	259467.3 (96.3)	2252846.0 (45.1)	117.46 [78.00, 209.47]
≥65	Male	Hispanic	1073657.5	384077.3 (39.1)	253283.3 (23.6)	88102.9 (8.2)	482695.8 (45.0)	781510.5 (81.3)	62885.8 (100.0)	294447.2 (27.4)	78.00 [49.50, 153.95]
<8	Female	Hispanic	2621972.2	4414.1 (0.2)	0.0 (NaN)	0.0 (NaN)	4492.5 (0.2)	0.0 (NaN)	0.0 (NaN)	459437.4 (17.5)	32.50 [21.50, 51.00]
8-<18	Female	Hispanic	3364517.3	367889.3 (11.7)	0.0 (NaN)	0.0 (NaN)	42439.4 (1.3)	5402.5 (0.3)	17670.1 (100.0)	1395204.3 (41.5)	65.00 [38.86, 115.68]
18-<40	Female	Hispanic	6427826.5	2555060.9 (42.4)	9944.6 (0.2)	12411.9 (0.2)	290591.9 (4.5)	165652.3 (2.9)	122113.7 (78.8)	2536176.1 (39.5)	94.76 [50.50, 162.57]
40-<65	Female	Hispanic	5073214.1	2407752.4 (50.4)	185353.8 (3.7)	96424.1 (1.9)	1111117.7 (21.9)	1053361.7 (22.4)	193346.6 (100.0)	1802254.0 (35.5)	78.00 [49.50, 131.77]
≥65	Female	Hispanic	1476989.6	628337.4 (47.2)	230217.9 (15.6)	71699.1 (4.9)	573609.4 (38.8)	991301.1 (74.6)	64017.4 (100.0)	310728.0 (21.0)	52.89 [37.00, 86.61]
<8	Male	Other	1248036.7	0.0 (0.0)	0.0 (NaN)	0.0 (NaN)	0.0 (0.0)	0.0 (NaN)	0.0 (NaN)	156943.5 (12.6)	41.98 [26.22, 78.98]
8-<18	Male	Other	1624875.4	145064.5 (9.5)	0.0 (NaN)	0.0 (NaN)	6017.1 (0.4)	2247.4 (0.3)	7975.0 (100.0)	564411.0 (34.7)	110.60 [74.50, 156.67]
18-<40	Male	Other	3242312.1	935488.4 (30.8)	14921.6 (0.5)	9507.3 (0.3)	87902.1 (2.7)	444013.9 (15.5)	93442.2 (90.5)	1050384.9 (32.4)	128.20 [65.83, 235.97]

40-<65	Male	Other	3512835.5	901922.7 (26.5)	303749.9 (8.6)	123420.4 (3.5)	846566.6 (24.1)	1195267.0 (37.2)	294279.9 (100.0)	693945.1 (19.8)	144.99 [78.00, 222.05]
≥65	Male	Other	1173025.6	229907.0 (22.4)	369142.1 (31.5)	138328.4 (11.8)	477085.7 (40.7)	881788.4 (84.5)	80466.7 (100.0)	176580.9 (15.1)	78.00 [51.76, 155.97]
<8	Female	Other	1261005.6	3744.1 (0.4)	0.0 (NaN)	0.0 (NaN)	0.0 (0.0)	0.0 (NaN)	0.0 (NaN)	162114.4 (12.9)	38.68 [23.00, 69.92]
8-<18	Female	Other	1527558.5	95447.3 (6.6)	0.0 (NaN)	0.0 (NaN)	15155.6 (1.0)	14446.3 (2.0)	7167.7 (100.0)	509147.0 (33.3)	68.50 [42.75, 124.31]
18-<40	Female	Other	3679371.3	1043904.0 (30.1)	22765.7 (0.7)	8193.5 (0.2)	150422.7 (4.1)	121478.8 (3.8)	25112.5 (100.0)	928052.2 (25.2)	81.69 [49.50, 199.21]
40-<65	Female	Other	3434224.2	1018888.5 (31.3)	231131.5 (6.7)	85978.2 (2.5)	520375.3 (15.2)	937380.3 (30.2)	154221.5 (78.4)	755878.3 (22.0)	78.00 [53.94, 170.59]
≥65	Female	Other	1244053.8	308812.7 (27.4)	150745.1 (12.1)	124517.9 (10.0)	431342.7 (34.7)	898822.9 (83.1)	59094.3 (100.0)	193920.0 (15.6)	76.40 [37.21, 142.45]

Note: Estimates apply survey weights to adjust for sampling and non-response, and to provide representative prevalence estimates for the civilian, non-institutionalized US population. IQR = interquartile range. NaN = not a number, zero prevalence. Data are available at: https://www.cdc.gov/nchs/nhanes/index.htm.
