# Supplementary material for: Evaluation of the sugar-sweetened beverage tax in Oakland, United States, 2015–2019: A quasi-experimental and cost-effectiveness study
Source: PLoS Med. 2023 Apr 18;20(4):e1004212. doi: 10.1371/journal.pmed.1004212 (PMC10112812; doi:10.1371/journal.pmed.1004212)
Supplement: S8 Table — (PDF) [file pmed.1004212.s011.pdf]

S8 Table. Estimates of outcome event costs, $US2021 from the Medical Expenditure Panel Survey (2018)

Condition	Insurance	Mean	SE
Cerebrovascular accident	Commercial	9862.67	3998.06
Cerebrovascular accident	Medicaid	8443.28	3169.13
Cerebrovascular accident	Medicare	8493.52	1818.663
Cerebrovascular accident	Uninsured	221.61	110.75
Diabetes mellitus	Commercial	3488.73	286.45
Diabetes mellitus	Medicaid	4523.84	618.87
Diabetes mellitus	Medicare	3528.01	258.66
Diabetes mellitus	Uninsured	1496.02	298.51
Dental caries and Periodontal disease	Commercial	320.33	99.79
Dental caries and Periodontal disease	Medicaid	339.86	83.68
Dental caries and Periodontal disease	Medicare	131.76	39.55
Dental caries and Periodontal disease	Uninsured	61.33	14.33
Coronary Heart disease	Commercial	4677.02	809.62
Coronary Heart disease	Medicaid	4689.59	871.04
Coronary Heart disease	Medicare	4959.37	541.24
Coronary Heart disease	Uninsured	2650.37	1207.23
Chronic Kidney Disease	Commercial	5453.22	921.40
Chronic Kidney Disease	Medicaid	14863.73	7312.74
Chronic Kidney Disease	Medicare	5965.20	848.78
Chronic Kidney Disease	Uninsured	3951.42	1654.73

Obesity costs were obtained separately, as they were not available from MEPS.(31) Data are available at: https://meps.ahrq.gov/mepstrends/hc_use/.
