# Supplementary material for: Evaluation of the sugar-sweetened beverage tax in Oakland, United States, 2015–2019: A quasi-experimental and cost-effectiveness study
Source: PLoS Med. 2023 Apr 18;20(4):e1004212. doi: 10.1371/journal.pmed.1004212 (PMC10112812; doi:10.1371/journal.pmed.1004212)
Supplement: S9 Table — (PDF) [file pmed.1004212.s012.pdf]

S9 Table. Incidence rates for simulated outcome events per Global Burden of Disease estimates (2019).

Age	Sex	Cause	Median estimate	Upper bound	Lower bound
1 to 4	Female	Chronic kidney disease	47.20894	63.01139	32.95862
1 to 4	Male	Chronic kidney disease	78.99168	104.5315	56.81939
1 to 4	Female	Diabetes mellitus	20.75532	33.99979	10.88079
1 to 4	Male	Diabetes mellitus	20.92664	34.58714	10.79071
1 to 4	Female	Ischemic heart disease	0	0	0
1 to 4	Male	Ischemic heart disease	0	0	0
1 to 4	Female	Obesity	29.15143	62.70151	8.883834
1 to 4	Male	Obesity	49.60735	103.7432	16.28102
1 to 4	Female	Oral disorders	60616.46	85643.84	36045.08
1 to 4	Male	Oral disorders	60472.97	84873.88	36643.87
1 to 4	Female	Stroke	14.7675	23.53738	8.533803
1 to 4	Male	Stroke	11.60042	18.70713	6.348078
10 to 14	Female	Chronic kidney disease	8.935179	18.85123	2.032214
10 to 14	Male	Chronic kidney disease	17.92176	31.54406	6.35788
10 to 14	Female	Diabetes mellitus	24.71004	36.33271	11.6812
10 to 14	Male	Diabetes mellitus	28.0946	41.34588	14.07157
10 to 14	Female	Ischemic heart disease	0	0	0
10 to 14	Male	Ischemic heart disease	0	0	0
10 to 14	Female	Obesity	48.0502	99.46208	16.8743
10 to 14	Male	Obesity	61.94173	134.5511	20.02888
10 to 14	Female	Oral disorders	58165.68	86585.52	33260.63
10 to 14	Male	Oral disorders	56314.47	84118.39	32086.81
10 to 14	Female	Stroke	12.46067	20.95553	6.978269
10 to 14	Male	Stroke	9.689149	17.22999	5.20904
15 to 19	Female	Chronic kidney disease	9.312343	19.63295	2.340498

15 to 19	Male	Chronic kidney disease	13.11725	25.46584	3.792602
15 to 19	Female	Diabetes mellitus	81.28169	111.9502	56.87148
15 to 19	Male	Diabetes mellitus	76.24342	104.2857	53.23194
15 to 19	Female	Ischemic heart disease	6.738575	12.79629	2.099048
15 to 19	Male	Ischemic heart disease	5.54222	11.17131	1.264462
15 to 19	Female	Obesity	36.35167	75.73231	12.36953
15 to 19	Male	Obesity	33.50627	69.64593	11.99161
15 to 19	Female	Oral disorders	53442.27	65117.43	39458.07
15 to 19	Male	Oral disorders	52953.88	65395.84	38967.05
15 to 19	Female	Stroke	11.6885	19.15019	6.232929
15 to 19	Male	Stroke	8.610932	14.09044	4.776862
20 to 24	Female	Chronic kidney disease	12.78041	23.48506	3.497487
20 to 24	Male	Chronic kidney disease	8.85641	18.8223	1.823736
20 to 24	Female	Diabetes mellitus	83.51173	124.0199	53.18252
20 to 24	Male	Diabetes mellitus	70.13368	103.5001	45.07453
20 to 24	Female	Ischemic heart disease	22.84585	31.90886	15.14046
20 to 24	Male	Ischemic heart disease	20.01754	28.67701	12.7714
20 to 24	Female	Obesity	319.6809	489.6904	187.0662
20 to 24	Male	Obesity	190.0897	309.966	97.45622
20 to 24	Female	Oral disorders	64100	71063.97	54353.67
20 to 24	Male	Oral disorders	62956.77	70608.28	52408.48
20 to 24	Female	Stroke	13.39925	20.65397	8.141808
20 to 24	Male	Stroke	9.437633	15.1512	5.64858
25 to 29	Female	Chronic kidney disease	19.17608	35.2521	5.678536
25 to 29	Male	Chronic kidney disease	8.265678	18.50378	1.385844
25 to 29	Female	Diabetes mellitus	136.598	189.6236	94.24653
25 to 29	Male	Diabetes mellitus	120.2759	172.6556	76.25104
25 to 29	Female	Ischemic heart	41.58277	58.0336	25.92408

		disease			
25 to 29	Male	Ischemic heart disease	37.88329	53.8894	22.98071
25 to 29	Female	Obesity	423.4328	629.1861	260.8128
25 to 29	Male	Obesity	274.3605	431.4326	156.4331
25 to 29	Female	Oral disorders	60677.54	69133.11	49694.49
25 to 29	Male	Oral disorders	58759.64	67637.61	48456.19
25 to 29	Female	Stroke	17.59288	28.20378	10.80187
25 to 29	Male	Stroke	12.16924	19.53653	7.274862
30 to 34	Female	Chronic kidney disease	29.10616	49.51797	14.11802
30 to 34	Male	Chronic kidney disease	10.75848	22.84361	2.140379
30 to 34	Female	Diabetes mellitus	237.47	312.4934	172.7902
30 to 34	Male	Diabetes mellitus	278.6054	354.9032	207.2591
30 to 34	Female	Ischemic heart disease	44.9364	60.69347	29.5394
30 to 34	Male	Ischemic heart disease	51.06009	68.33908	36.34178
30 to 34	Female	Obesity	568.3644	863.5027	340.1502
30 to 34	Male	Obesity	409.1734	629.6077	227.9775
30 to 34	Female	Oral disorders	56671.45	66598.18	46235.83
30 to 34	Male	Oral disorders	53635.46	62868.61	44273.73
30 to 34	Female	Stroke	24.82795	36.03912	17.23565
30 to 34	Male	Stroke	17.67038	25.48621	12.15957
35 to 39	Female	Chronic kidney disease	54.11553	84.83986	29.1552
35 to 39	Male	Chronic kidney disease	28.49731	48.11333	10.9662
35 to 39	Female	Diabetes mellitus	347.1296	464.0388	248.5495
35 to 39	Male	Diabetes mellitus	496.9561	627.1345	377.4229
35 to 39	Female	Ischemic heart disease	32.90723	46.25885	22.36828
35 to 39	Male	Ischemic heart disease	59.54823	83.35469	41.27403
35 to 39	Female	Obesity	790.3325	1131.882	509.8727
35 to 39	Male	Obesity	674.4296	1006.209	411.476

35 to 39	Female	Oral disorders	53835.62	63594.06	42960.55
35 to 39	Male	Oral disorders	50671.37	59676.49	41521.49
35 to 39	Female	Stroke	35.10436	50.17962	23.29063
35 to 39	Male	Stroke	25.94098	35.96924	17.91387
40 to 44	Female	Chronic kidney disease	125.569	183.1872	79.66411
40 to 44	Male	Chronic kidney disease	85.5683	125.144	51.99416
40 to 44	Female	Diabetes mellitus	453.8418	567.6665	351.9388
40 to 44	Male	Diabetes mellitus	674.3721	816.9709	552.2411
40 to 44	Female	Ischemic heart disease	37.35114	50.96383	26.13758
40 to 44	Male	Ischemic heart disease	91.22844	116.2314	70.35777
40 to 44	Female	Obesity	1011.055	1431.915	663.6449
40 to 44	Male	Obesity	963.4911	1387.441	583.4624
40 to 44	Female	Oral disorders	51358.48	61564.78	39617.99
40 to 44	Male	Oral disorders	47858.33	57814.51	38417.01
40 to 44	Female	Stroke	53.22779	69.96869	39.60985
40 to 44	Male	Stroke	40.99572	53.20251	31.27122
45 to 49	Female	Chronic kidney disease	292.0827	406.9746	197.3051
45 to 49	Male	Chronic kidney disease	178.2918	252.4696	117.1585
45 to 49	Female	Diabetes mellitus	555.7975	745.4559	392.3488
45 to 49	Male	Diabetes mellitus	809.5076	1080.436	579.6102
45 to 49	Female	Ischemic heart disease	58.26768	79.27672	41.82589
45 to 49	Male	Ischemic heart disease	146.0978	189.8235	108.4931
45 to 49	Female	Obesity	1294.029	1829.539	844.0946
45 to 49	Male	Obesity	1316.065	1870.693	830.753
45 to 49	Female	Oral disorders	48583.64	59817.3	38623.21
45 to 49	Male	Oral disorders	44793.61	55174.57	35374.85
45 to 49	Female	Stroke	79.1976	109.9172	56.22762
45 to 49	Male	Stroke	62.83415	87.48028	45.85873

5 to 9	Female	Chronic kidney disease	14.47226	25.13897	5.476308
5 to 9	Male	Chronic kidney disease	27.85626	44.10313	13.74514
5 to 9	Female	Diabetes mellitus	27.4092	40.97809	14.08947
5 to 9	Male	Diabetes mellitus	28.381	43.3426	15.29218
5 to 9	Female	Ischemic heart disease	0	0	0
5 to 9	Male	Ischemic heart disease	0	0	0
5 to 9	Female	Obesity	52.69883	110.589	17.42904
5 to 9	Male	Obesity	67.33227	143.2751	22.18554
5 to 9	Female	Oral disorders	134659.7	187609.9	79935.93
5 to 9	Male	Oral disorders	133358.7	184383.5	80173.74
5 to 9	Female	Stroke	13.76285	22.26023	7.743933
5 to 9	Male	Stroke	10.9155	18.36201	5.884777
50 to 54	Female	Chronic kidney disease	491.102	699.3977	343.0563
50 to 54	Male	Chronic kidney disease	292.5233	425.7128	190.679
50 to 54	Female	Diabetes mellitus	779.489	962.2528	616.8167
50 to 54	Male	Diabetes mellitus	1100.585	1343.236	889.426
50 to 54	Female	Ischemic heart disease	90.5885	113.802	71.54553
50 to 54	Male	Ischemic heart disease	234.1449	285.9085	189.3745
50 to 54	Female	Obesity	1729.658	2440.044	1155.129
50 to 54	Male	Obesity	1760.98	2542.411	1085.876
50 to 54	Female	Oral disorders	44531.91	54871.86	35010.22
50 to 54	Male	Oral disorders	40928.34	50730.66	31854.11
50 to 54	Female	Stroke	115.1498	146.5021	87.98533
50 to 54	Male	Stroke	96.85711	121.0435	76.38754
55 to 59	Female	Chronic kidney disease	757.9621	1090.676	474.1046
55 to 59	Male	Chronic kidney disease	540.8723	783.8166	332.9661
55 to 59	Female	Diabetes mellitus	1096.24	1402.18	854.4132

55 to 59	Male	Diabetes mellitus	1492.396	1913.406	1104.479
55 to 59	Female	Ischemic heart disease	134.3119	182.8444	96.75124
55 to 59	Male	Ischemic heart disease	355.357	472.9829	265.7716
55 to 59	Female	Obesity	2224.892	3153.262	1461.071
55 to 59	Male	Obesity	2335.267	3364.514	1405.036
55 to 59	Female	Oral disorders	40365.35	50334.37	31153.53
55 to 59	Male	Oral disorders	37009.85	45936.54	29048.8
55 to 59	Female	Stroke	161.0831	213.0763	118.1743
55 to 59	Male	Stroke	143.063	189.7126	105.3147
60 to 64	Female	Chronic kidney disease	1263.884	1778.07	857.4195
60 to 64	Male	Chronic kidney disease	1044.279	1493.417	712.9018
60 to 64	Female	Diabetes mellitus	1033.917	1311.153	819.2947
60 to 64	Male	Diabetes mellitus	1419.718	1768.158	1083.539
60 to 64	Female	Ischemic heart disease	228.3792	287.4071	183.3736
60 to 64	Male	Ischemic heart disease	554.7294	680.1519	456.7813
60 to 64	Female	Obesity	2826.694	4039.145	1828.655
60 to 64	Male	Obesity	3073.417	4445	1783.624
60 to 64	Female	Oral disorders	37297.69	49073.43	27492.02
60 to 64	Male	Oral disorders	34096.73	43115.18	25827.52
60 to 64	Female	Stroke	228.4677	289.793	177.297
60 to 64	Male	Stroke	204.8995	257.597	164.0091
65 to 69	Female	Chronic kidney disease	1840.512	2523.201	1265.698
65 to 69	Male	Chronic kidney disease	1673.625	2300.536	1114.962
65 to 69	Female	Diabetes mellitus	681.8848	847.6414	539.9686
65 to 69	Male	Diabetes mellitus	1037.656	1288.914	785.7609
65 to 69	Female	Ischemic heart disease	372.7657	473.2158	284.2389
65 to 69	Male	Ischemic heart disease	832.1809	1034.929	638.32

65 to 69	Female	Obesity	3310.444	4792.469	2051.895
65 to 69	Male	Obesity	3613.702	5387.327	2129.278
65 to 69	Female	Oral disorders	35266.39	46434.05	26618.54
65 to 69	Male	Oral disorders	32282.92	41028.81	24486.15
65 to 69	Female	Stroke	317.2954	439.1914	223.1179
65 to 69	Male	Stroke	282.3612	385.7272	201.4039
70 to 74	Female	Chronic kidney disease	2449.708	3376.374	1609.801
70 to 74	Male	Chronic kidney disease	2310.376	3171.246	1499.789
70 to 74	Female	Diabetes mellitus	398.4208	570.5591	243.3962
70 to 74	Male	Diabetes mellitus	652.3497	905.8562	423.4187
70 to 74	Female	Ischemic heart disease	616.384	726.3197	513.2555
70 to 74	Male	Ischemic heart disease	1153.543	1371.445	950.782
70 to 74	Female	Obesity	3634.091	5194.613	2240.017
70 to 74	Male	Obesity	3802.381	5754.806	2137.334
70 to 74	Female	Oral disorders	32956.16	43891.03	24071.31
70 to 74	Male	Oral disorders	30345.42	38246.21	22633.78
70 to 74	Female	Stroke	435.5593	565.887	330.4449
70 to 74	Male	Stroke	363.2235	469.4101	279.274
75 to 79	Female	Chronic kidney disease	3181.87	4578.244	2201.394
75 to 79	Male	Chronic kidney disease	2806.927	4016.569	1967.205
75 to 79	Female	Diabetes mellitus	161.5537	220.9683	107.4764
75 to 79	Male	Diabetes mellitus	245.0214	323.5711	168.5994
75 to 79	Female	Ischemic heart disease	959.1368	1209.919	740.4364
75 to 79	Male	Ischemic heart disease	1518.773	1885.523	1190.596
75 to 79	Female	Obesity	3661.655	5402.555	2143.973
75 to 79	Male	Obesity	3654.51	5730.531	1931.247
75 to 79	Female	Oral disorders	30517	40709.2	22284.27
75 to 79	Male	Oral disorders	28323.04	36280.88	20725.01

75 to 79	Female	Stroke	583.2417	788.762	421.1265
75 to 79	Male	Stroke	447.4865	588.7487	330.398
80 plus	Female	Chronic kidney disease	2311.544	2955.68	1806.494
80 plus	Male	Chronic kidney disease	2440.764	3138.82	1898.697
80 plus	Female	Diabetes mellitus	26.88573	38.61053	17.95358
80 plus	Male	Diabetes mellitus	29.3942	43.5164	19.02187
80 plus	Female	Ischemic heart disease	1774.115	2055.552	1527.685
80 plus	Male	Ischemic heart disease	2378.043	2786.306	2042.609
80 plus	Female	Obesity	3422.67	5292.809	1812.941
80 plus	Male	Obesity	2971.46	4979.208	1442.115
80 plus	Female	Oral disorders	25071.72	33382.98	17889.81
80 plus	Male	Oral disorders	23915.98	31464.81	16765.27
80 plus	Female	Stroke	1098.833	1366.989	880.1213
80 plus	Male	Stroke	731.8555	885.9343	593.19

Note: Rates as estimates for the state of California, and are listed per 100,000 persons. Obesity rates are expressed in years of life with disability per incident case to account for variable disability by body mass index level, while others are expressed as event rates with the disutility estimate for each year after onset listed in the main text. Data are available at: http://ghdx.healthdata.org/gbd-results-tool.
