# Supplementary material for: Evaluation of the sugar-sweetened beverage tax in Oakland, United States, 2015–2019: A quasi-experimental and cost-effectiveness study
Source: PLoS Med. 2023 Apr 18;20(4):e1004212. doi: 10.1371/journal.pmed.1004212 (PMC10112812; doi:10.1371/journal.pmed.1004212)
Supplement: S10 Table — (PDF) [file pmed.1004212.s013.pdf]

**S10 Table.** Mortality rates for simulated outcome events per Global Burden of Disease estimates (2019)

| Age | Sex | Cause | Median value | Upper bound | Lower bound |
|---|---|---|---|---|---|
| 1 to 4 | Male | Diabetes mellitus | 0.040336 | 0.065674 | 0.026369 |
| 1 to 4 | Female | Diabetes mellitus | 0.034134 | 0.060811 | 0.021914 |
| 5 to 9 | Male | Diabetes mellitus | 0.026485 | 0.04553 | 0.016257 |
| 5 to 9 | Female | Diabetes mellitus | 0.028333 | 0.05223 | 0.018501 |
| 10 to 14 | Male | Diabetes mellitus | 0.071645 | 0.107934 | 0.046178 |
| 10 to 14 | Female | Diabetes mellitus | 0.062548 | 0.104181 | 0.040312 |
| 15 to 19 | Male | Diabetes mellitus | 0.21292 | 0.28931 | 0.150547 |
| 15 to 19 | Female | Diabetes mellitus | 0.149579 | 0.203974 | 0.107335 |
| 20 to 24 | Male | Diabetes mellitus | 0.568084 | 0.743835 | 0.42981 |
| 20 to 24 | Female | Diabetes mellitus | 0.356182 | 0.513922 | 0.244755 |
| 25 to 29 | Male | Diabetes mellitus | 1.002082 | 1.28192 | 0.790172 |
| 25 to 29 | Female | Diabetes mellitus | 0.692095 | 0.988016 | 0.452787 |
| 30 to 34 | Male | Diabetes mellitus | 1.742084 | 2.225743 | 1.334054 |
| 30 to 34 | Female | Diabetes mellitus | 1.082318 | 1.532216 | 0.720855 |
| 35 to 39 | Male | Diabetes mellitus | 2.867417 | 3.73508 | 2.144957 |
| 35 to 39 | Female | Diabetes mellitus | 1.511755 | 2.145911 | 1.057804 |
| 40 to 44 | Male | Diabetes mellitus | 5.117566 | 6.874634 | 3.700818 |
| 40 to 44 | Female | Diabetes mellitus | 2.69438 | 3.759992 | 1.881682 |
| 45 to 49 | Male | Diabetes mellitus | 8.798182 | 11.94687 | 6.290751 |
| 45 to 49 | Female | Diabetes mellitus | 4.485769 | 6.258539 | 3.158869 |

| | | | | | |
|---|---|---|---|---|---|
| 50 to 54 | Male | Diabetes mellitus | 14.86757 | 20.97037 | 10.55405 |
| 50 to 54 | Female | Diabetes mellitus | 8.15874 | 11.50065 | 5.714527 |
| 55 to 59 | Male | Diabetes mellitus | 25.22204 | 34.50475 | 17.35855 |
| 55 to 59 | Female | Diabetes mellitus | 13.20807 | 18.49208 | 9.390755 |
| 60 to 64 | Male | Diabetes mellitus | 38.74198 | 52.88457 | 26.58489 |
| 60 to 64 | Female | Diabetes mellitus | 21.52354 | 29.61189 | 15.10305 |
| 65 to 69 | Male | Diabetes mellitus | 54.51901 | 75.75144 | 37.85474 |
| 65 to 69 | Female | Diabetes mellitus | 30.97836 | 41.81911 | 22.23016 |
| 70 to 74 | Male | Diabetes mellitus | 79.74812 | 111.1633 | 55.92012 |
| 70 to 74 | Female | Diabetes mellitus | 48.96432 | 65.63482 | 35.01432 |
| 75 to 79 | Male | Diabetes mellitus | 114.5758 | 156.2393 | 81.17738 |
| 75 to 79 | Female | Diabetes mellitus | 74.25793 | 102.2763 | 52.82016 |
| 80 plus | Male | Diabetes mellitus | 222.555 | 273.9504 | 175.1378 |
| 80 plus | Female | Diabetes mellitus | 170.0434 | 214.5504 | 127.5119 |
| 1 to 4 | Male | Chronic kidney disease | 0.062131 | 0.088122 | 0.04319 |
| 1 to 4 | Female | Chronic kidney disease | 0.068327 | 0.096874 | 0.048951 |
| 5 to 9 | Male | Chronic kidney disease | 0.029602 | 0.040236 | 0.021264 |
| 5 to 9 | Female | Chronic kidney disease | 0.029728 | 0.041987 | 0.021042 |
| 10 to 14 | Male | Chronic kidney disease | 0.031233 | 0.042138 | 0.022737 |
| 10 to 14 | Female | Chronic kidney disease | 0.027258 | 0.039036 | 0.018921 |
| 15 to 19 | Male | Chronic kidney disease | 0.084855 | 0.124473 | 0.056966 |
| 15 to 19 | Female | Chronic kidney disease | 0.084066 | 0.121298 | 0.058049 |

| | | | | | |
|---|---|---|---|---|---|
| 20 to 24 | Male | Chronic kidney disease | 0.239823 | 0.338567 | 0.169125 |
| 20 to 24 | Female | Chronic kidney disease | 0.195708 | 0.290904 | 0.127146 |
| 25 to 29 | Male | Chronic kidney disease | 0.561692 | 0.783777 | 0.400491 |
| 25 to 29 | Female | Chronic kidney disease | 0.446805 | 0.651999 | 0.285503 |
| 30 to 34 | Male | Chronic kidney disease | 1.148726 | 1.573335 | 0.803241 |
| 30 to 34 | Female | Chronic kidney disease | 1.007521 | 1.483115 | 0.64278 |
| 35 to 39 | Male | Chronic kidney disease | 2.209701 | 3.039342 | 1.594967 |
| 35 to 39 | Female | Chronic kidney disease | 1.475727 | 2.085291 | 1.003527 |
| 40 to 44 | Male | Chronic kidney disease | 4.099358 | 5.594324 | 2.877357 |
| 40 to 44 | Female | Chronic kidney disease | 2.470011 | 3.50169 | 1.65756 |
| 45 to 49 | Male | Chronic kidney disease | 6.714295 | 9.189574 | 4.535006 |
| 45 to 49 | Female | Chronic kidney disease | 4.232985 | 5.926607 | 2.886339 |
| 50 to 54 | Male | Chronic kidney disease | 12.25863 | 17.16636 | 8.304177 |
| 50 to 54 | Female | Chronic kidney disease | 7.452955 | 10.31271 | 5.250545 |
| 55 to 59 | Male | Chronic kidney disease | 20.14723 | 28.37828 | 14.16215 |
| 55 to 59 | Female | Chronic kidney disease | 13.72148 | 18.93504 | 9.610233 |
| 60 to 64 | Male | Chronic kidney disease | 33.70424 | 45.83913 | 23.53394 |
| 60 to 64 | Female | Chronic kidney disease | 22.96455 | 31.25505 | 16.57105 |
| 65 to 69 | Male | Chronic kidney disease | 50.76519 | 68.77506 | 35.32945 |
| 65 to 69 | Female | Chronic kidney disease | 36.51747 | 48.50581 | 26.35829 |
| 70 to 74 | Male | Chronic kidney disease | 82.74838 | 109.5951 | 59.55005 |
| 70 to 74 | Female | Chronic kidney disease | 62.15792 | 83.27555 | 46.37783 |

| | | | | | |
|---|---|---|---|---|---|
| 75 to 79 | Male | Chronic kidney disease | 142.8786 | 189.1261 | 102.6906 |
| 75 to 79 | Female | Chronic kidney disease | 103.1054 | 137.8639 | 75.59011 |
| 80 plus | Male | Chronic kidney disease | 437.0099 | 530.2135 | 352.6953 |
| 80 plus | Female | Chronic kidney disease | 321.7972 | 404.6999 | 240.9206 |
| 15 to 19 | Male | Ischemic heart disease | 0.347747 | 0.491701 | 0.232738 |
| 15 to 19 | Female | Ischemic heart disease | 0.146875 | 0.191751 | 0.105502 |
| 20 to 24 | Male | Ischemic heart disease | 0.964134 | 1.369089 | 0.668135 |
| 20 to 24 | Female | Ischemic heart disease | 0.317896 | 0.435726 | 0.219207 |
| 25 to 29 | Male | Ischemic heart disease | 1.896095 | 2.514761 | 1.397496 |
| 25 to 29 | Female | Ischemic heart disease | 0.650719 | 0.925283 | 0.450309 |
| 30 to 34 | Male | Ischemic heart disease | 4.175796 | 5.5737 | 3.049957 |
| 30 to 34 | Female | Ischemic heart disease | 1.446188 | 1.984362 | 1.012857 |
| 35 to 39 | Male | Ischemic heart disease | 8.610621 | 11.17408 | 6.505735 |
| 35 to 39 | Female | Ischemic heart disease | 2.600626 | 3.46391 | 1.88949 |
| 40 to 44 | Male | Ischemic heart disease | 19.59986 | 25.20264 | 14.66429 |
| 40 to 44 | Female | Ischemic heart disease | 5.516207 | 7.435796 | 3.905718 |
| 45 to 49 | Male | Ischemic heart disease | 38.79531 | 50.12696 | 28.8965 |
| 45 to 49 | Female | Ischemic heart disease | 10.48783 | 13.942 | 7.758319 |
| 50 to 54 | Male | Ischemic heart disease | 76.87931 | 101.1162 | 57.81341 |
| 50 to 54 | Female | Ischemic heart disease | 22.88489 | 30.14044 | 17.2928 |
| 55 to 59 | Male | Ischemic heart disease | 130.144 | 167.1298 | 97.16508 |
| 55 to 59 | Female | Ischemic heart disease | 40.28583 | 52.78425 | 30.1914 |

| | | | | | |
|---|---|---|---|---|---|
| 60 to 64 | Male | Ischemic heart disease | 211.3481 | 269.6928 | 160.5224 |
| 60 to 64 | Female | Ischemic heart disease | 75.03201 | 94.99677 | 57.91595 |
| 65 to 69 | Male | Ischemic heart disease | 301.917 | 382.4433 | 231.5722 |
| 65 to 69 | Female | Ischemic heart disease | 124.5548 | 155.2939 | 98.51318 |
| 70 to 74 | Male | Ischemic heart disease | 469.8261 | 584.2528 | 372.2573 |
| 70 to 74 | Female | Ischemic heart disease | 223.919 | 281.9131 | 174.2769 |
| 75 to 79 | Male | Ischemic heart disease | 750.3705 | 926.6438 | 586.0144 |
| 75 to 79 | Female | Ischemic heart disease | 404.4416 | 498.5174 | 322.0388 |
| 80 plus | Male | Ischemic heart disease | 2432.225 | 2906.511 | 1976.835 |
| 80 plus | Female | Ischemic heart disease | 2044.595 | 2505.119 | 1549.032 |
| 1 to 4 | Male | Stroke | 0.115287 | 0.18249 | 0.068657 |
| 1 to 4 | Female | Stroke | 0.121673 | 0.184266 | 0.072921 |
| 5 to 9 | Male | Stroke | 0.091151 | 0.138474 | 0.055434 |
| 5 to 9 | Female | Stroke | 0.094621 | 0.146886 | 0.052685 |
| 10 to 14 | Male | Stroke | 0.151081 | 0.233942 | 0.086839 |
| 10 to 14 | Female | Stroke | 0.125508 | 0.193874 | 0.075153 |
| 15 to 19 | Male | Stroke | 0.343403 | 0.567928 | 0.187496 |
| 15 to 19 | Female | Stroke | 0.300312 | 0.457615 | 0.18506 |
| 20 to 24 | Male | Stroke | 0.611519 | 1.01205 | 0.344863 |
| 20 to 24 | Female | Stroke | 0.410641 | 0.653551 | 0.241133 |
| 25 to 29 | Male | Stroke | 1.00732 | 1.53879 | 0.569008 |
| 25 to 29 | Female | Stroke | 0.79008 | 1.191702 | 0.484057 |
| 30 to 34 | Male | Stroke | 1.814214 | 2.907445 | 1.094202 |
| 30 to 34 | Female | Stroke | 1.41378 | 2.067474 | 0.887581 |
| 35 to 39 | Male | Stroke | 3.611402 | 5.352099 | 2.242009 |
| 35 to 39 | Female | Stroke | 2.352558 | 3.402194 | 1.483638 |
| 40 to 44 | Male | Stroke | 6.507712 | 9.547401 | 4.260317 |

| | | | | | |
|---|---|---|---|---|---|
| 40 to 44 | Female | Stroke | 4.702547 | 6.529345 | 3.335933 |
| 45 to 49 | Male | Stroke | 10.63263 | 15.97021 | 6.823049 |
| 45 to 49 | Female | Stroke | 7.518618 | 10.63386 | 4.966157 |
| 50 to 54 | Male | Stroke | 17.47402 | 24.80922 | 11.84883 |
| 50 to 54 | Female | Stroke | 12.37809 | 17.31848 | 8.291398 |
| 55 to 59 | Male | Stroke | 27.99712 | 40.32742 | 19.10914 |
| 55 to 59 | Female | Stroke | 18.44641 | 25.78309 | 12.4342 |
| 60 to 64 | Male | Stroke | 43.77145 | 63.14011 | 30.30574 |
| 60 to 64 | Female | Stroke | 28.35967 | 40.43065 | 19.14916 |
| 65 to 69 | Male | Stroke | 66.01193 | 92.43327 | 44.88107 |
| 65 to 69 | Female | Stroke | 45.6302 | 62.46927 | 31.79161 |
| 70 to 74 | Male | Stroke | 116.9031 | 160.4849 | 82.55467 |
| 70 to 74 | Female | Stroke | 91.17465 | 120.4542 | 66.42132 |
| 75 to 79 | Male | Stroke | 215.2479 | 286.4262 | 151.8393 |
| 75 to 79 | Female | Stroke | 184.7408 | 245.9961 | 132.7514 |
| 80 plus | Male | Stroke | 710.6714 | 867.2281 | 558.5492 |
| 80 plus | Female | Stroke | 884.325 | 1096.817 | 662.0541 |
| 1 to 4 | Male | Obesity | 0.010739 | 0.021672 | 0.003777 |
| 1 to 4 | Female | Obesity | 0.007408 | 0.015824 | 0.002206 |
| 5 to 9 | Male | Obesity | 0.018676 | 0.036417 | 0.006495 |
| 5 to 9 | Female | Obesity | 0.014262 | 0.027185 | 0.005093 |
| 10 to 14 | Male | Obesity | 0.031584 | 0.06104 | 0.01208 |
| 10 to 14 | Female | Obesity | 0.018552 | 0.037798 | 0.006592 |
| 15 to 19 | Male | Obesity | 0.029426 | 0.054378 | 0.01074 |
| 15 to 19 | Female | Obesity | 0.016311 | 0.032092 | 0.005791 |
| 20 to 24 | Male | Obesity | 1.774075 | 2.695118 | 0.971489 |
| 20 to 24 | Female | Obesity | 0.963971 | 1.402468 | 0.616555 |
| 25 to 29 | Male | Obesity | 3.391178 | 4.813618 | 1.957047 |
| 25 to 29 | Female | Obesity | 1.780967 | 2.509562 | 1.120434 |
| 30 to 34 | Male | Obesity | 7.011468 | 9.946243 | 4.41936 |
| 30 to 34 | Female | Obesity | 3.521976 | 4.851746 | 2.384132 |
| 35 to 39 | Male | Obesity | 14.12321 | 19.44924 | 9.100937 |

| | | | | | |
|---|---|---|---|---|---|
| 35 to 39 | Female | Obesity | 6.293325 | 8.665221 | 4.32177 |
| 40 to 44 | Male | Obesity | 27.08885 | 37.74227 | 16.99738 |
| 40 to 44 | Female | Obesity | 11.73409 | 15.95854 | 8.1266 |
| 45 to 49 | Male | Obesity | 50.82813 | 71.63687 | 32.17153 |
| 45 to 49 | Female | Obesity | 21.79361 | 29.42136 | 15.12241 |
| 50 to 54 | Male | Obesity | 89.5342 | 126.8544 | 56.12834 |
| 50 to 54 | Female | Obesity | 47.75555 | 66.13284 | 33.01107 |
| 55 to 59 | Male | Obesity | 139.6835 | 203.4705 | 83.5627 |
| 55 to 59 | Female | Obesity | 74.55196 | 102.7312 | 51.30366 |
| 60 to 64 | Male | Obesity | 214.684 | 305.8116 | 128.8031 |
| 60 to 64 | Female | Obesity | 119.9958 | 163.8699 | 80.45587 |
| 65 to 69 | Male | Obesity | 278.4678 | 402.5292 | 157.202 |
| 65 to 69 | Female | Obesity | 168.8785 | 231.2627 | 111.3921 |
| 70 to 74 | Male | Obesity | 359.3239 | 543.4621 | 190.0174 |
| 70 to 74 | Female | Obesity | 250.7915 | 349.0283 | 156.5294 |
| 75 to 79 | Male | Obesity | 467.9001 | 733.3531 | 235.8288 |
| 75 to 79 | Female | Obesity | 369.6105 | 532.5744 | 225.5891 |
| 80 plus | Male | Obesity | 853.0226 | 1429.456 | 402.9665 |
| 80 plus | Female | Obesity | 1034.654 | 1597.27 | 546.4463 |

Note: Rates as estimates for the state of California, and are listed per 100,000 persons. Data are available at: http://ghdx.healthdata.org/gbd-results-tool.
