# Supplementary material for: Evaluation of the sugar-sweetened beverage tax in Oakland, United States, 2015–2019: A quasi-experimental and cost-effectiveness study
Source: PLoS Med. 2023 Apr 18;20(4):e1004212. doi: 10.1371/journal.pmed.1004212 (PMC10112812; doi:10.1371/journal.pmed.1004212)
Supplement: S1 Fig — (PDF) [file pmed.1004212.s014.pdf]

S1 Figure. Average monthly ounces per product-store sold of SSBs and untaxed beverages

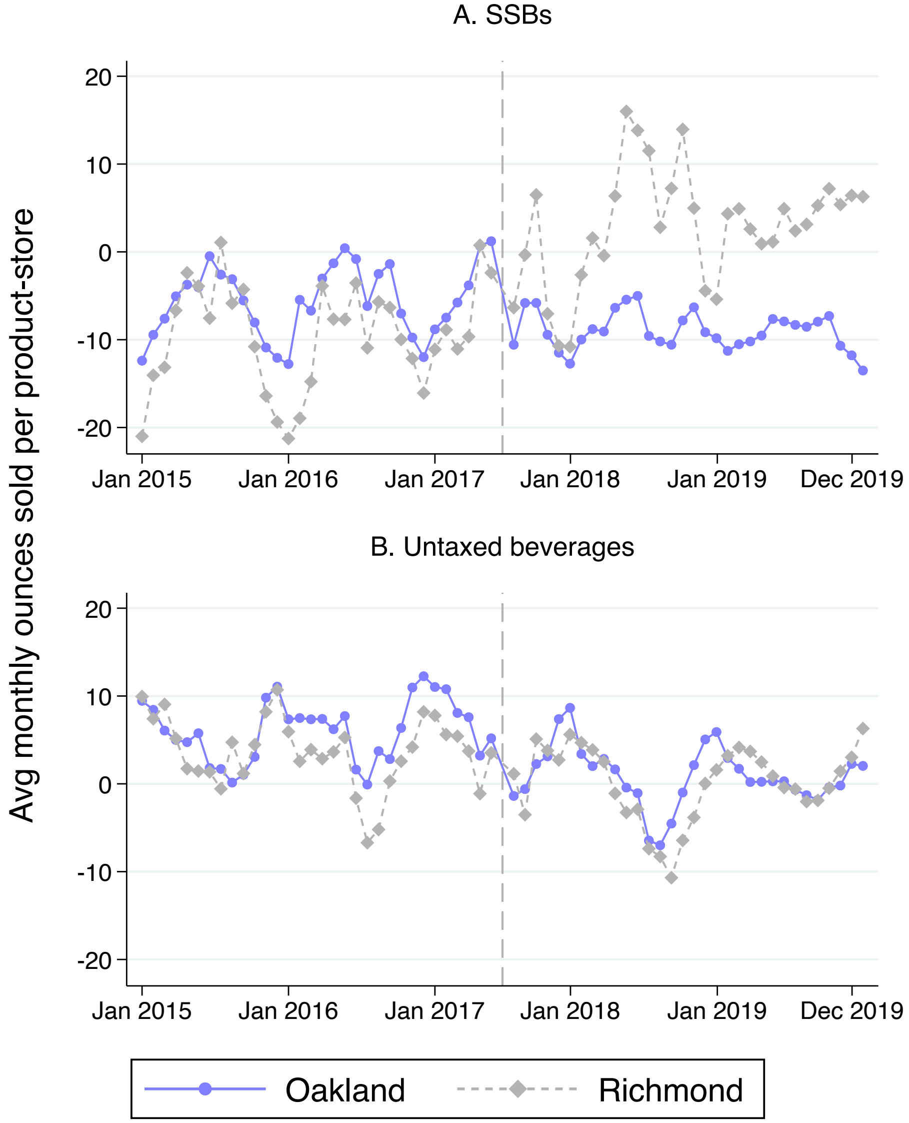

Note: This figure shows average monthly ounces per product-store sold of SSBs (Panel A) and untaxed beverages (Panel B) after partialing out UPC, store, and week fixed effects.
