# Supplementary material for: Evaluation of the sugar-sweetened beverage tax in Oakland, United States, 2015–2019: A quasi-experimental and cost-effectiveness study
Source: PLoS Med. 2023 Apr 18;20(4):e1004212. doi: 10.1371/journal.pmed.1004212 (PMC10112812; doi:10.1371/journal.pmed.1004212)
Supplement: S8 Fig — (PDF) [file pmed.1004212.s021.pdf]

S8 Figure. Time-varying association between the Oakland tax and volume sales of sweet snacks (plots from event study difference-in-differences models)

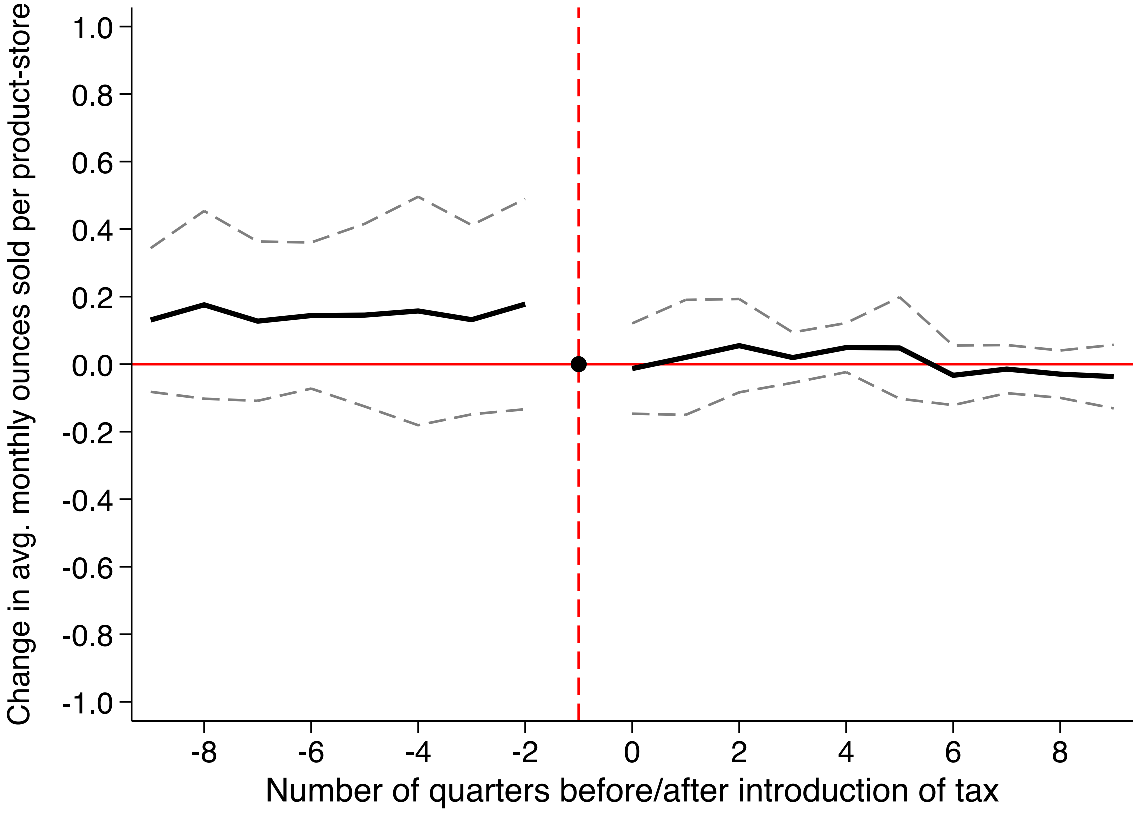

Note: Sweet snacks includes cookies and doughnuts (8,507 unique UPCs). Quarterly estimates are relative to the quarter pre-tax (quarter -1, red dotted line). Black dotted lines are 95% confidence intervals from robust standard errors clustered by zip code.
