# Supplementary figures and images for: Evaluation of the sugar-sweetened beverage tax in Oakland, United States, 2015–2019: A quasi-experimental and cost-effectiveness study
Source: PLoS Med. 2023 Apr 18;20(4):e1004212. doi: 10.1371/journal.pmed.1004212 (PMC10112812; doi:10.1371/journal.pmed.1004212)

**S10 Figure.** Net present value of accrued disease-specific costs over time

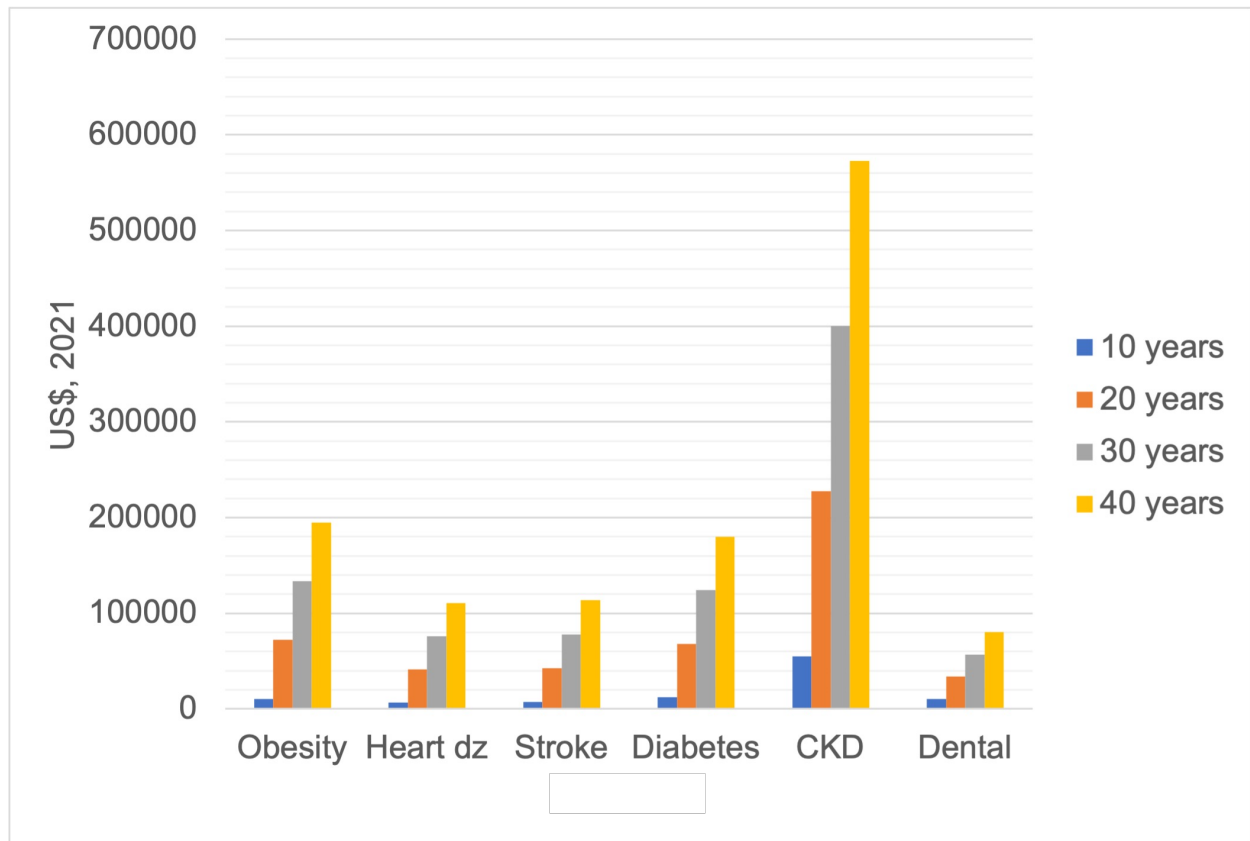

Supplement: S10 Fig — (PDF) [file pmed.1004212.s023.pdf]
